# PEPTIDE IDR-1002 REGULATES THE ANTIOXIDANT AND ANTI-INFLAMMATORY RESPONSES BY ACTIVATING THE KEAP1-NRF2 SIGNALING PATHWAY

**DOI:** 10.12688/f1000research.177148.1

**Published:** 2026-02-06

**Authors:** Marco Antonio Romero-Durán, María Cristina Maldonado-Pichardo, Jose Manuel Perez-Aguilar, Victor Manuel Baizabal Aguirre

**Affiliations:** 1 Centro Multidisciplinario de Estudios en Biotecnología, Facultad de Medicina Veterinaria y Zootecnia, Universidad Michoacana de San Nicolás de Hidalgo, Morelia, Michoacan, 58100, Mexico; 2 Centro Multidisciplinario de Estudios en Biotecnología, Facultad de Medicina Veterinaria y Zootecnia, Universidad Michoana de San Nicolás de Hidalgo, Morelia, Michoacan, 58100, Mexico; 3 School of Chemical Sciences, Meritorious Autonomous University of Puebla, Puebla, Puebla, 72570, Mexico; 4 Centro Multidisciplinario de Estudios en Biotecnología, Facultad de Medicina Veterinaria y Zootecnia, Universidad Michoacana de San Nicolás de Hidalgo, Morelia, Michoacan, 58100, Mexico

**Keywords:** IDR-1002, peptide, Nrf2, oxidative stress, inflammation, TNF-α.

## Abstract

**Background:**

Oxidative stress and inflammation are mutually reinforcing physiological processes. The transcription factor Nrf2 (Nuclear factor erythroid 2-related factor 2) acts as the master regulator of redox homeostasis and anti-inflammatory signaling. To overcome the off-target limitations of conventional electrophilic Nrf2 activators, this study investigates selective peptide-based modulators. We present data indicating that an innate defense regulator (IDR)-1002 peptide is able to activate the Nrf2-mediated antioxidant response and mitigate inflammation in an endothelial cell model.

**Methods:**

We first screened several IDR peptides IDR-1 (Lys-Ser-Arg-Ile-Val-Pro-Ala-Ile-Pro-Val-Ser-Leu-Leu), IDR-1018 (Val-Arg-Leu-Ile-Val-Ala-Val-Arg-Ile-Trp-Arg-Arg), HH2 (Val-Gln-Leu-Arg-Ile-Arg-Val-Ala-Val-Ile-Arg-Ala) and IDR-1002 (Val-Gln-Arg-Trp-Leu-Ile-Val-Trp-Arg-Ile-Arg-Lys) for their ability to induce Nrf2 nuclear translocation in HEK293 and bovine endothelial cells (BEC) via Western blot and ELISA. Transcriptional activity was assessed in HepG2 cells using an ARE (Antioxidant Response Element)-luciferase reporter assay to determine the EC _50_. Downstream expression of antioxidant enzymes, including HO-1 (Heme Oxygenase-1), NQO1 (NAD(P)H:quinone oxidoreductase 1), and GCLM (Glutamate-Cysteine Ligase Modifier Subunit), was quantified by ELISA. GST (Glutathione S-Transferase) activity induced by IDR-1002 was measured using a CDNB-GSH conjugation assay. Finally, the functional capacity to reduce H _2_O _2_-induced ROS (Reactive Oxygen Species) and TNF-stimulated inflammation was measured in BECs.

**Results:**

Among the tested peptides, IDR-1002 induced the most potent concentration-dependent nuclear translocation of Nrf2 in both cell lines. IDR-1002 activated ARE-dependent transcription with an EC _50_ of 18.57 μM. This activation led to a significant time-dependent increase in HO-1, NQO1, and GCLM protein levels, alongside enhanced GST activity. Functionally, IDR-1002 pre-treatment resulted in a dose-dependent reduction of intracellular ROS (up to 5.4-fold at 50 μM) and a significant decrease in TNF-α expression in stimulated BECs.

**Conclusions:**

IDR-1002 acts as a potent dual-function regulator that simultaneously activates the Nrf2 antioxidant response and inhibits NF-κB-mediated inflammation. These findings highlight IDR-1002 as a promising therapeutic candidate for chronic diseases characterized by the interplay between oxidative stress and inflammation.

## Introduction

Reactive oxygen species (ROS) are essential signaling molecules that, at low concentrations, contribute to physiological cellular functions, including cell proliferation, differentiation, and host defense. However, excessive accumulation of ROS or reactive nitrogen species (RNS) disrupts redox homeostasis and induces oxidative stress, leading to damage of lipids, proteins, and DNA that may cause numerous chronic and acute diseases, including neurodegenerative disorders, cancer, cardiovascular diseases, and infections. ^1– 3^

The transcription factor nuclear factor erythroid 2–related factor 2 (Nrf2) is a central regulator of the cellular antioxidant response that orchestrates a protective gene expression program against oxidative and electrophilic stress. ^3– 5^ Under basal conditions, Nrf2 is sequestered in the cytoplasm by the Kelch-like ECH-associated protein 1 (Keap1), which targets it for ubiquitination and proteasomal degradation. Upon exposure to various stress agents, Nrf2 dissociates from Keap1 and translocates to the nucleus, where it binds to the antioxidant response element (ARE) and induces the transcription of phase II detoxification enzymes such as heme oxygenase-1 (HO-1), NAD(P) H quinone oxidoreductase 1 (NQO1), and glutamate–cysteine ligase modifier subunit (GCLM), among others. ^3, 6^

Due to its central role in cytoprotection, Nrf2 is currently a promising therapeutic target for treating diseases associated with oxidative stress and chronic inflammation. Several small-molecule Nrf2 activators, including sulforaphane, chalcone, and fumaric acid derivatives such as dimethyl fumarate (DMF), have been investigated in preclinical and clinical settings. These compounds typically act as electrophiles that modify cysteine residues (e.g., Cys151, Cys273, Cys288) on Keap1 to disrupt its interaction with Nrf2. ^3, 6, 7^ Although these molecules appear to have appropriate characteristics, they have been observed to alter the activity of other proteins bearing Cys residues. This may non-selectively modify other cysteine-containing proteins, leading to off-target effects. Because of these limitations, there is increasing interest in the development of selective peptide-based modulators that can disrupt the Nrf2–Keap1 interaction with higher specificity and fewer side effects. ^8^ Nevertheless, the ability of peptides to simultaneously activate Nrf2 signaling while inhibiting a pro-inflammatory response mediated by nuclear factor kappa B (NF-κB) remains underexplored. ^9– 12^

Interestingly, some natural peptides have shown dual regulatory functions by activating Nrf2 and concurrently inhibiting NF-κB-mediated inflammation. For instance, the decapeptide YD1 (Ala-Pro-Lys-Gly-Val-Gln-Gly-Pro-Asn-Gly) induces an increase in Nrf2 activity and expression of downstream antioxidants such as HO-1 and NQO1, while suppressing pro-inflammatory mediators through TLR4/MyD88/NF-κB pathway inhibition. ^11^ Similarly, peptides such as K-8-K (Lys-Val-Leu-Pro-Val-Pro-Gly-Lys), S-10-S (Ser-Leu-Val-Asn-Asn-Asp-Asp-Arg-Asp-Ser), and LP-5 (Leu-Pro-Val-Thr-Lys), derived from milk, soy, and walnuts, respectively, ^13, 14^ show comparable dual activity by promoting antioxidant enzyme expression and reducing inflammasome activation. These findings highlight that modulation of both oxidative stress and inflammation is an emerging characteristic among certain natural peptides. ^11^ Alternatively, synthetic peptides, derived from natural host peptides, has also demonstrated to be a valuable strategy. In this context, the innate defense regulator (IDR) anti-inflammatory peptides IDR-1 (Lys-Ser-Arg-Ile-Val-Pro-Ala-Ile-Pro-Val-Ser-Leu-Leu), ^15, 16^ IDR-1018 (Val-Arg-Leu-Ile-Val-Ala-Val-Arg-Ile-Trp-Arg-Arg), ^17^ HH2 (Val-Gln-Leu-Arg-Ile-Arg-Val-Ala-Val-Ile-Arg-Ala), ^17^ and IDR-1002 (Val-Gln-Arg-Trp-Leu-Ile-Val-Trp-Arg-Ile-Arg-Lys) ^17– 19^ emerge as good candidates to test their ability to modulate the activity of Nrf2.

In particular, IDR-1002 was identified from a library of bactenecin, an antimicrobial peptide found in bovine neutrophils, ^18^ as a peptide able to confer protection against invasive *Staphylococcus aureus* infection through chemokine induction. ^19^ Furthermore, IDR-1002 significantly reduced the production of reactive oxygen and nitrogen species (ROS/RNS) and attenuated inflammation and tissue damage *in vivo* in a mouse ear inflammation model. ^20^ Our group has also previously reported that IDR-1002 modulates inflammation in RAW 264.7 macrophages challenged with lipopolysaccharide (LPS), TNFα, or IL-1β via inhibition of IκBα phosphorylation and NF-κB p65 nuclear translocation. ^21^ Based on these findings, we hypothesized that IDR-1002 may also be able to upregulate an antioxidant response through Nrf2 signaling in addition to its known anti-inflammatory activity. Supporting this notion, another study using a chicken hepatocyte non-parenchymal cell co-culture showed that IDR-1002 reduces pro-inflammatory cytokine release while increasing Nrf2 production, highlighting its dual role in regulating inflammation and antioxidant responses; however, definitive proof of its direct effects on Nrf2 activation and subsequently the downstream antioxidant enzyme expression was not assessed. ^12^

Thus, in this study, we present experimental evidence on the potential of IDR-1002 to modulate Nrf2-mediated antioxidant responses in human embryonic kidney (HEK293) cells and bovine endothelial cells (BEC). Our data demonstrate that IDR-1002 induces Nrf2 nuclear translocation, enhances ARE-driven transcriptional activity, and upregulates expression of the key antioxidant enzymes HO-1 and NQO1, and enhances Glutathione S-Transferase (GST) activity. Besides, IDR-1002 reduced ROS production in H _2_O _2_-stimulated cells and attenuated TNFα expression in TNFα-stimulated BECs. Together, these results highlight the dual regulatory potential of IDR-1002 on redox and inflammatory pathways and support it as a potential therapeutic peptide that selectively modulates both the Nrf2 and NF-κB signaling axes.

## Materials and methods

### Peptide synthesis

The immunomodulatory peptides IDR-1018 (VRLIVAVRIWRR-NH2), IDR-HH2 (VQLRIRVAVIRA-NH2), IDR-1 (KSRIVPAI-PVSLL-NH2), and IDR-1002, (VQRWLIVWRIRK-NH2) with the C-terminal amidated were synthesized by solid-phase Fmoc chemistry (CPC Scientific, USA) and obtained at a purity greater than 95%.

### Antibodies and reagents

Primary and secondary antibodies used: mouse β-actin (sc-47778), laminin (sc-7293), GAPDH-IgG (sc-32233), and anti-mouse IgG-BP-HRP (sc-525409) (Santa Cruz Biotechnology, USA); Histone H3 Rabbit (9717), rabbit NQO1 (62262S), and anti-rabbit IgG-HRP (7074S) (Cell Signaling Technology, USA); rabbit Nrf2 (ADI-KAP-TF125) and HO-1 (ADI-OSA-150-F) (Enzo Life Sciences, USA). Additional reagents included non-fat dry milk (Bio-Rad, USA), Luminol (Millipore, USA), protease and phosphatase inhibitor cocktail (cOmplete™, Roche, Switzerland), tert-butyl hydroperoxide (TBHP; Sigma-Aldrich, cat. 458139), ferrous sulfate (FeSO _4_; cat. 7782-63-0), D, L-sulforaphane (SFN; cat. S4441), Tris-HCl, NaCl, 1-chloro-2,4-dinitrobenzene (CDNB), hydrogen peroxide (H _2_O _2_; cat. 7722-84-1), DCFDA (cat. 4091-99-0), Igepal CA-930, Na-pyrophosphate, NaF, Na-orthovanadate, RIPA buffer (cat. R0278), and chemiluminescent substrate (Millipore, USA). TNFα was purchased from (ACROBiosystem-Switzerland; cat. TNA-H4211). Cell culture media and supplements were obtained from BPS Bioscience (USA), including MEM, Thaw Medium 1, 1K nutrient medium, fetal bovine serum (FBS), non-essential amino acids, sodium pyruvate, penicillin/streptomycin, geneticin (cat. 79533), and Luciferase ONE-Step reagent (cat. 60690). Trypsin-EDTA 1X (cat. T2601), L-glutathione reduced (gsh, cat. G4251), Bradford Reagent (cat. B6916) and MTT (3-(4,5 dimethylthiazol-2-yl)-2,5-diphenyltetrazolium bromide (cat. 475989) were purchased from Sigma-Aldrich.

### Cell culture

ARE-Hep-G2 cells (Hep-G2 cells) (BPS Bioscience, cat. 60513) which express luciferase under control of ARE sequences were cultured in 1K medium containing DMEM supplemented with 10% FBS, 1% non-essential amino acids, 1 mM sodium pyruvate, 1% penicillin/streptomycin, and 600 μg/mL geneticin at 37°C in a humidified atmosphere with 5% CO _2_. Cells between passages 8–23 were used. BEC cells (bovine umbilical vein endothelial cells) were kindly provided by Dr. Carmen Clapp from the Institute of Neurobiology, National Autonomous University of Mexico (UNAM). These cells were immortalized by transfection with HPV16 E6E7 oncogenes, that extend their replicative lifespan of primary BEC from 40 to more than 120 passages without signs of senescence. Importantly, BEC cells retain key endothelial characteristics, including the uptake of acetylated low-density lipoproteins, von Willebrand factor expression, specific lectin binding, and proliferative responses to vascular endothelial growth factor. ^22^ HEK-293 cells were purchased from the American Type Culture Collection (ATCC; cat. CRL-1573). HEK-293 and BEC cells were cultured in DMEM supplemented with 10% FBS, 10,000 U/mL penicillin, and 1 mg/mL streptomycin, at 37°C in 5% CO _2_. Cells between passages 8 and 20 were used.

### Luciferase reporter assay

Hep-G2 cells were plated at 40,000 cells/well in 96-well plates with 45 μL of 1K medium without geneticin. Cells were treated with 5 μl of IDR-1002 (final concentration: 0.5–300 μM) or FeSO _4_ (positive control; final concentration: 100 μM). After 8–10 h incubation at 37°C, 100 μL of Luciferase ONE-Step reagent was added and the plate was shaken for 15 min. Luminescence was measured using a Varioskan ^TM^ LUX plate reader (Thermo Fisher Scientific, USA). Fold induction was calculated by normalizing to untreated controls. TBHP, FeSO _4_, or SFN were used as Nrf2-activators.

### MTT cell viability assay

Hep-G2 and BEC cells were seeded in 96-well plates at 5 x 10 ^4^ cells/mL and incubated for 24 h at 37 ^°^C. Cells were treated with IDR-1002 (1–100 μM) or TBHP (10 μM) for 8 h. Then, 50 μL of 10% MTT solution was added, and incubation continued for 4 h. Medium was removed and formazan crystals were solubilized in 100% DMSO. Absorbance was read at 570 nm using a Varioskan ^TM^ LUX reader.

### Intracellular ROS quantification

ROS levels were measured in BEC cells using the DCFDA (2’,7’-dichlorofluorescein diacetate) fluorescence assay. ^23^ Cells were pretreated with IDR-1002 (1-50 μM) for 4 h, followed by incubation with 30 μM DCFDA at 37 °C for 30 min. Cells were then washed with PBS and stimulated with 50 μM H _2_O _2_ for 20 min. Fluorescence was measured at excitation/emission wavelengths of 485/530 nm using a Varioskan ^TM^ LUX reader.

### Protein extraction and Western blotting

BEC cells were grown in 6-well plates to ~90% confluence, serum-starved for ≥ 4 h, and then treated with 1, 10, 25, or 50 μM IDR-1002 for 1 h or 4 h before lysis for subsequent Western blots of Nrf2, HO-1, and NQO1. Total protein (cytosolic plus nuclear from control and treated cells) was extracted by washing cells with cold PBS and lysing them with 80 μL of a cold buffer containing 20 mM Tris–HCl pH 7.5, 150 mM NaCl, 1% Igepal CA-930, 10 mM Na-pyrophosphate, and 50 mM NaF supplemented with 1 mM Na-orthovanadate and 1x protease inhibitors. Lysates were centrifuged at 13,000 × g (20 min, 4°C), and supernatants collected and transferred to ice-cold Eppendorf tubes. Fractions (Cytoplasmic and Nuclear) were prepared using 80 μL of RIPA buffer and sonicated with a Qsonica Q125 sonicator (Newtown, USA) adjusted to 25% amplitude for a total of 15 s (3 × 5 s, with 7 s rest in between). Protein concentration was measured by the Bradford method using BSA as standard. Samples (50-60 μg) were separated by 10% SDS-PAGE and transferred in a wet chamber to 0.22 μm nitrocellulose membranes for 1 h at 250–300 mA. Detection was performed using appropriate antibodies and Immobilon HRP chemiluminescent kit. Imaging was done with the LI-COR Odyssey system.

### Nrf2 and antioxidant enzymes quantification

Levels of Nrf2, HO-1, NQO1 and GCLM were measured using competitive ELISA kits (MyBioSource, USA), according to the manufacturer’s instructions.

### Glutathione S-transferase activity

BEC cells were treated with 50 μM IDR-1002 for 6, 12, 18, or 24 h. Cell lysates were prepared in Tris-HCl buffer (50 mM, pH 7.4) containing 150 mM NaCl and protease inhibitors. GST activity was determined by measuring the absorbance at 340 nm of the 1-chloro-2,4-dinitrobenzene assay (CDNB)-glutathione (GSH) conjugation assay.

### TNFα quantification

TNFα levels in BEC cells supernatants were measured by ELISA per the manufacturer’s protocol (MyBioSource, USA).

### Statistical analysis

All data are presented as mean ± standard deviation (SD). Analyses were conducted using GraphPad Prism 8 (GraphPad Software, USA). Unpaired Student’s t test was used for two-group comparisons while one-way ANOVA with Tukey’s post hoc test was applied for multiple comparisons. Statistical significance is indicated by * *P* < 0.05, ** *P* < 0.01, *** *P* < 0.001, **** *P* < 0.0001; ns = not significant and ND = not detected.

## Results

### IDR peptides promote Nrf2 nuclear translocation in HEK293 cells

**Figure 1.**
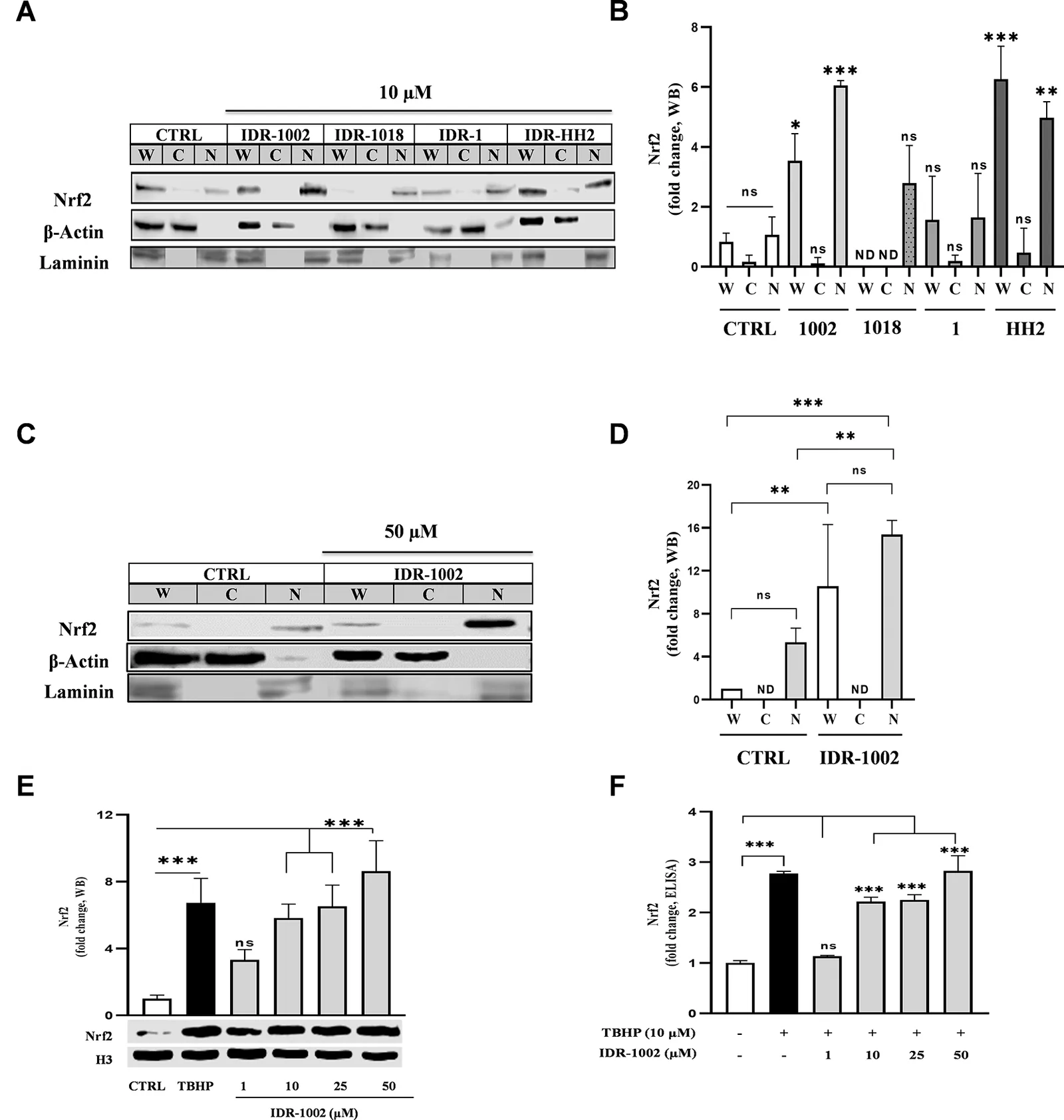
Nrf2 nuclear translocation induced by IDR peptides in HEK293 cells and by IDR-1002 in bovine endothelial cells (BEC cells). ( **A, C**) Western blot detection showing the relative abundance of the Nrf2 protein (MW ~100 kDa) in whole (W), cytoplasmic (C), and nuclear (N) protein enriched extracts from HEK293 cells. Cells were treated for 1 h with 10 μM IDR-1002, IDR-1018, IDR-1, and IDR-HH2 or with 50 μM IDR-1002. β-Actin (MW ~42 kDa) and laminin (MW ~70 kDa) were used as loading controls for the cytoplasmic and nuclear extracts, respectively. **(B, D)** Nrf2 nuclear accumulation is expressed as fold changes normalized to the untreated control (CTRL). **(E)** Representative Western blot showing nuclear Nrf2 levels in BEC cells treated for 1 h with 1, 10, 25, and 50 μM IDR-1002 and a positive control, tert-butyl hydroperoxide (TBHP, 10 μM). The graph above the blot shows the densitometric analysis of the relative fold change in nuclear Nrf2 levels, normalized to the untreated control (CTRL). Histone H3 (H3, 17 kDa) was used as a nuclear loading control. **(F)** Quantification of nuclear Nrf2 levels by competitive ELISA in BEC cells treated under the same conditions as in (E). Mean values for IDR-1002 at 10, 25, and 50 μM, as well as TBHP, were significantly increased compared to CTRL. Comparisons not indicated by asterisks were not statistically significant (ns) or not detect (ND). These results are representative of three independent experiments (n = 3). Bars indicate the mean ± standard deviation (SD). Asterisks indicate a statistically significant difference, determined by two-way ANOVA, followed by Tukey's post hoc test with the following significance levels: *p < 0.05, **p < 0.01 and ***p < 0.001.

Several synthetic protein-protein interaction (PPI) inhibitors of Keap1-Nrf2 have been reported to activate the Nrf2 pathway; however, their use is limited by low selectivity and cytotoxicity effects. ^24, 25^ In this context, IDR peptides have emerged as promising alternatives due to their immunomodulatory properties and low cytotoxic potential. ^17, 26– 28^ Notably, among the IDR peptides group, IDR-1002 exhibits a potent inhibitory NF-κB activity. ^1,^ ^21^ Thus, we initially explored the ability of IDR-1002 and three other IDR peptides, namely IDR-1018, IDR-HH2, and IDR-1, to activate Nrf2 nuclear translocation. HEK293 cells were treated with 10 μM of each peptide and Nrf2 abundance was assessed in whole-cell, cytoplasmic, and nuclear fractions by Western blot ( Figure 1A, B). Among all peptides tested, IDR-1002 induced the strongest nuclear translocation of Nrf2. IDR-HH2 also promoted Nrf2 nuclear accumulation, albeit to a lesser extent, while IDR-1 had minimal effect. To confirm the dose-dependency of IDR-1002, increasing concentrations (up to 50 μM) were tested ( Figure 1C, D). A 15-fold increase in nuclear Nrf2 abundance was observed at 50 μM compared to a 6-fold increase at 10 μM, supporting IDR-1002 as the most potent Nrf2 activator among the tested peptides. These data suggest that sequence variations between IDR peptides influence their ability to modulate the Nrf2 signaling pathway. Based on these results and those we previously reported on NF-κB inhibition, ^21^ IDR-1002 was selected for subsequent experiments.

### IDR-1002 activates Nrf2 nuclear translocation in BEC cells

Next, to determine whether IDR-1002 activates Nrf2 in other cell types, BEC cells were treated with increasing concentrations of the peptide. Given the central role of endothelial cells in redox signaling and inflammation, and the antagonistic interplay between NF-κB and Nrf2 in vascular homeostasis, ^19, 29– 32^ BEC cells are considered a relevant model. IDR-1002 induced a concentration-dependent increase in Nrf2 nuclear translocation ( Figure 1E). Also, the amount of Nrf2 measured in BEC cells lysates by ELISA increased with IDR-1002 treatment from 10 to 50 μM ( Figure 1F). These data indicate that IDR-1002 activated Nrf2 nuclear translocation in BEC cells.

### IDR-1002 induces Nrf2 transcriptional activity

**Figure 2.**
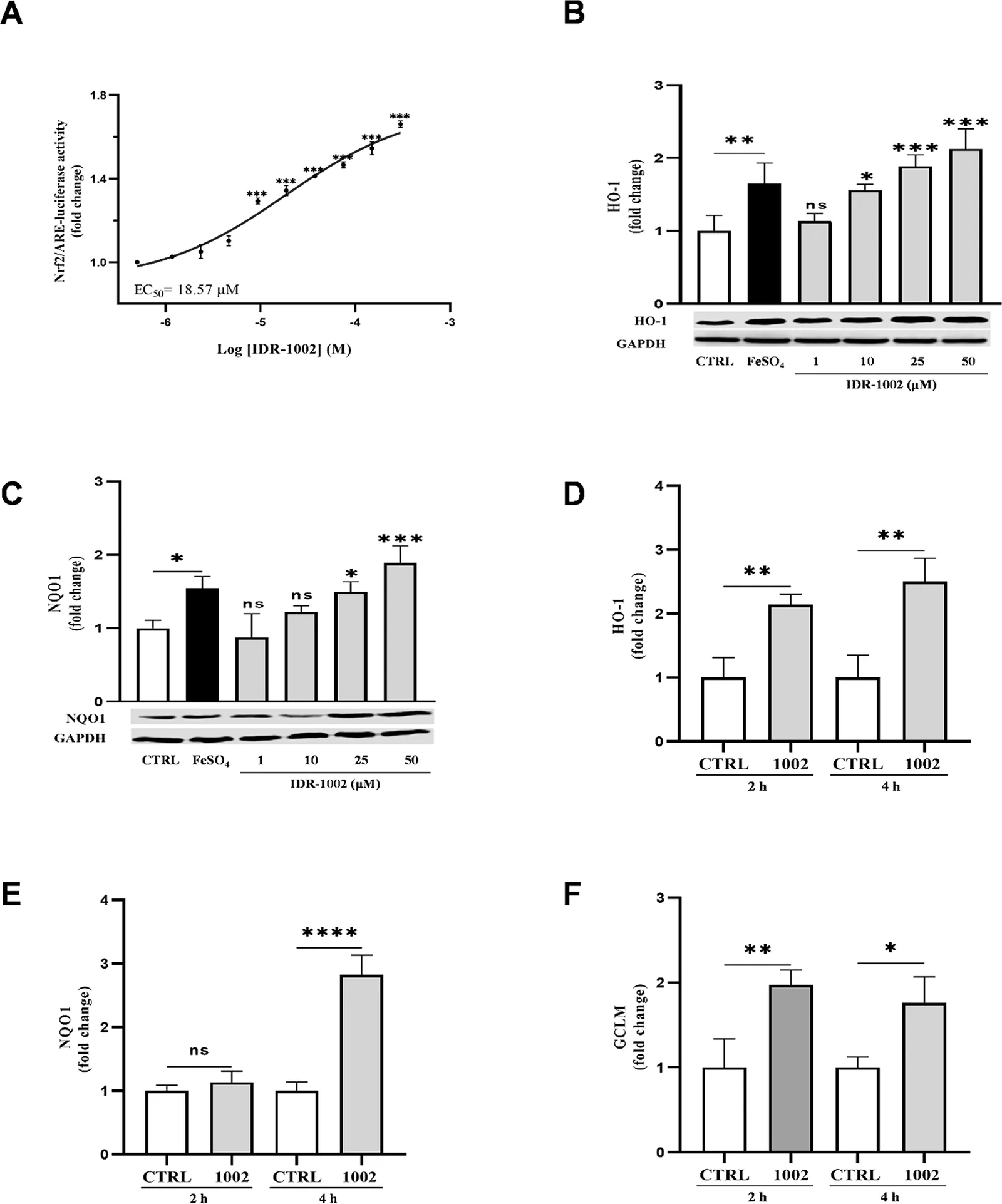
IDR-1002 activates the Nrf2 transcriptional pathway and downstream phase II antioxidant enzymes in BEC cells. **(A)** Measurement of Nrf2-mediated transcriptional activity in HepG2-ARE-luciferase reporter cells treated with IDR-1002 (0.5 to 300 μM) for 8 h. Activity is measured as a fold change in luminescence relative to the untreated control, resulting in an EC _50_ of 18.57 μM. **(B, C)** Western blot analysis of the protein levels of the antioxidant enzymes HO-1 (28 kDa) and NQO1 (29 kDa) in BEC cells treated for 4 h with IDR-1002 1, 10, 25, and 50 μM. Glyceraldehyde-3-phosphate dehydrogenase (GAPDH, 37 kDa) served as a loading control for both blots. Iron (II) sulfate (FeSO _4_, 150 μM) was used as a positive control. The graph above the blot represents the densitometric analysis of the relative fold change in HO-1 expression, normalized to the unstimulated control. Mean values for HO-1 at 10, 25, and 50 μM IDR-1002, and those for NQO1 at 25 and 50 μM IDR-1002 were significantly different from the control group. **(D, E, F)** ELISA quantification of the protein levels of the antioxidant enzymes, HO-1 **(D),** NQO1 **(E),** and GCLM **(F)** in BEC cells treated with 50 μM of IDR-1002 for 2 or 4 h. Protein expression was normalized to total protein content and compared to the respective time-matched untreated control (CTRL 2 h or CTRL 4 h). Treatment with IDR-1002 significantly increased the expression of these antioxidant enzymes in a time-dependent manner. Comparisons not indicated by asterisks were not statistically significant (ns) or not detect (ND). These results are representative of three independent experiments (n = 3). Bars indicate the mean ± standard deviation (SD). Asterisks indicate a statistically significant difference, determined by two-way ANOVA, followed by Tukey's post hoc test with the following significance levels: *p < 0.05, **p < 0.01 and *** *p* < 0.001.

Under basal conditions, Nrf2 is retained in the cytoplasm by the kelch domain of Keap1. Upon activation, Nrf2 dissociates from Keap1, translocates to the nucleus and binds to ARE in target genes promoters, driving the expression of antioxidant genes. ^33– 35^ To evaluate the Nrf2 transcriptional activity Hep-G2 cells stably expressing the ARE-luciferase reporter, were treated with IDR-1002. A concentration-dependent increase in ARE-luciferase activity was observed with an EC _50_ of 18.57 μM ( Figure 2A). Importantly, cell viability assays confirmed that IDR-1002 did not induce cytotoxicity neither in Hep-G2 or BEC cells at 1, 10, 25, 50, or 100 μM ( **Extended data Figure E1**).

### IDR-1002 induces the expression of antioxidant enzymes

The Nrf2-dependent phase II antioxidant enzymes HO-1, NQO-1 and GCLM, mitigate oxidative stress and can suppress NF-κB activity. ^7, 36, 37^ Western blot analysis confirmed the increased expression of HO-1 and NQO1 following IDR-1002 treatment ( Figure 2B, C) supporting its role in Nrf2 activation and downstream antioxidant gene induction. This was further corroborated by ELISA-based quantification of HO-1, NQO1 and GCLM, at 2- and 4-h post-treatment with 50 μM IDR-1002, demonstrating time-dependent upregulation of these enzymes ( Figure 2D, E, F).

### IDR-1002 induces glutathione S-transferase activity

**Figure 3.**
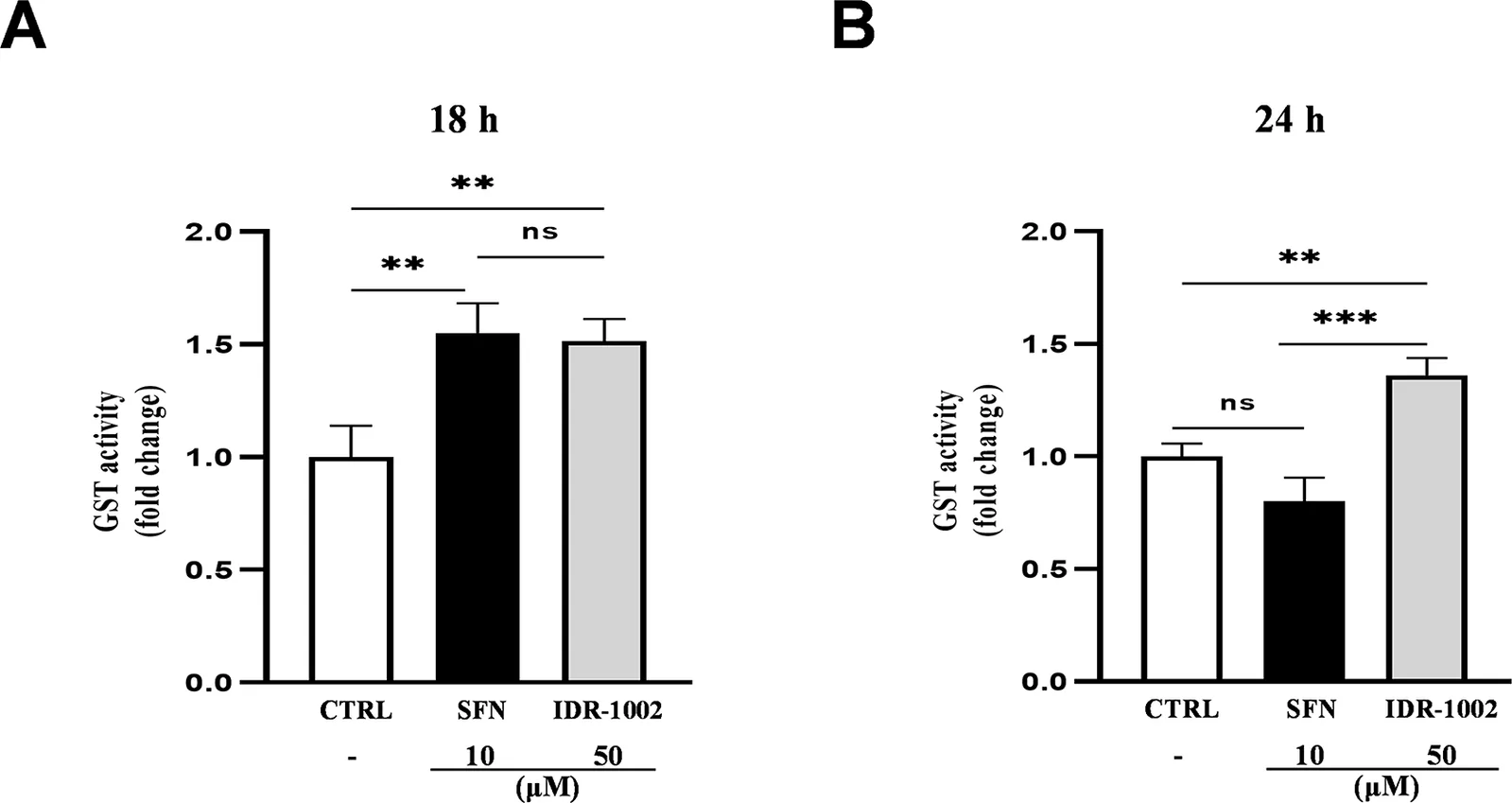
Induction of GST activity by IDR-1002 in BEC cells. BEC cells were treated with 50 μM IDR-1002 for 18 ( **A**) and 24 h ( **B**). Sulforaphane (SFN, 10 μM) was used as a positive control. Glutathione S-transferase (GST) activity was quantified after each treatment using the 1-chloro-2,4-dinitrobenzene (CDNB) colorimetric assay. IDR-1002-stimulated cells showed a significant increase in GST activity compared to the untreated control. Data are representative of three independent experiments (n = 3) and are presented as the mean ± standard deviation (SD). Statistical significance was determined by the two-way ANOVA with Tukey’s post hoc test. Asterisks indicate significance levels as follows: *p < 0.05, **p < 0.01, and ***p < 0.001.

Glutathione S-transferases (GSTs) are a family of phase II detoxifying enzymes that catalyze the conjugation of glutathione (GSH) to reactive intermediates, thus protecting against oxidative stress. ^38^ To determine whether IDR-1002 activates GSTs, BEC cells were treated with 50 μM IDR-1002 for 18, and 24 h. GST activity increased significantly at 18 h ( Figure 3A), with a no significant slight reduction at 24 h ( Figure 3B), compared with the untreated control for each time point. No GST activity was detected at 6 and 12 h (data not shown). These results suggest that IDR-1002 contributes to cellular protection by inducing GST-mediated detoxification responses.

### IDR-1002 reduces intracellular ROS production

**Figure 4.**
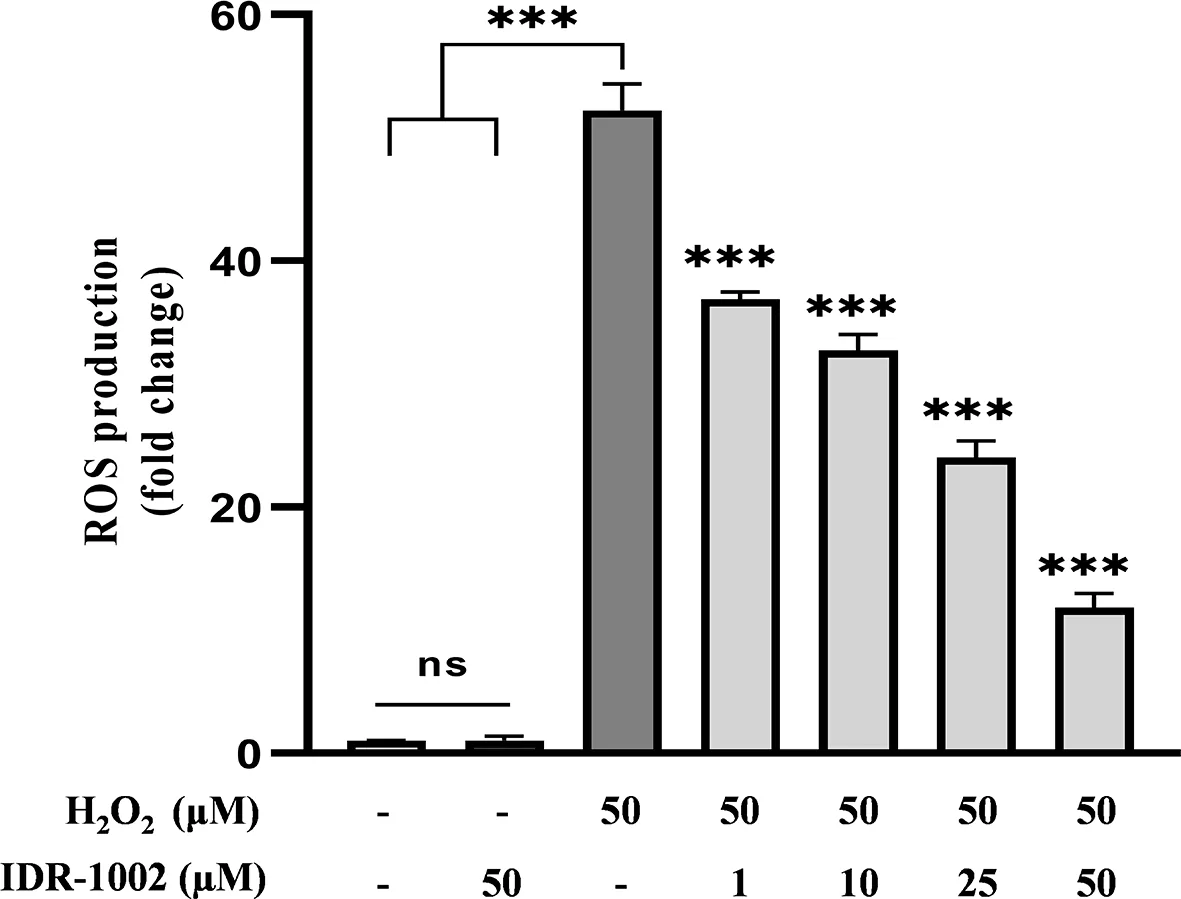
IDR-1002 reduces ROS production in H _2_O _2_-stimulated BEC cells. BEC cells were pretreated with 1, 10, 25, and 50 μM IDR-1002 for 4 h and subsequently incubated with 50 μM H _2_O _2_ for 15 min to induce oxidative stress. Intracellular ROS production was then quantified using the 2',7'-dichlorofluorescin diacetate (DCFH-DA) fluorescence assay. Data are representative of three independent experiments (n = 3) and are presented as the mean ± standard deviation (SD). Asterisks indicate a statistical difference compared to the H _2_O _2_-treated control, with a significance level of ***p < 0.001.

To assess the antioxidant effect of IDR-1002, intracellular ROS levels were measured in BEC cells treated with H _2_O _2_ following IDR-1002 pre-treatment. BEC cells were pre-incubated with the indicated concentrations of the peptide for 4 h and subsequently incubated with 50 μM H _2_O _2_ for 15 min. Basal ROS levels remained unchanged in both control and in the presence of 50 μM IDR-1002-treated cells, indicating that IDR-1002 did not exert pro-oxidant effects ( Figure 4). In H _2_O _2_-stimulated cells, treatment with IDR-1002 resulted in a dose-dependent reduction in ROS levels. At 25 and 50 μM, IDR-1002 decreased ROS levels by approximately 2.4- and 5.4-fold respectively, compared to the H _2_O _2_-treated control. These findings are consistent with previous studies reporting that 18 μM IDR-1002 reduces H _2_O _2_-induced ROS production by 1.6-fold. ^12^ Altogether, these results indicate that IDR-1002 exerts its antioxidant effects through the activation of the Nrf2 pathway.

### IDR-1002 reduces TNFα expression

Chronic oxidative stress is closely linked to inflammation, and TNFα is a key cytokine involved in various redox-related pathologies, including cancer, Alzheimer’s, Parkinson’s, diabetes, Crohn’s, and cardiovascular disorders. ^39– 44^ ROS can induce TNFα production, and elevated TNFα can further amplify ROS levels.

**Figure 5.**
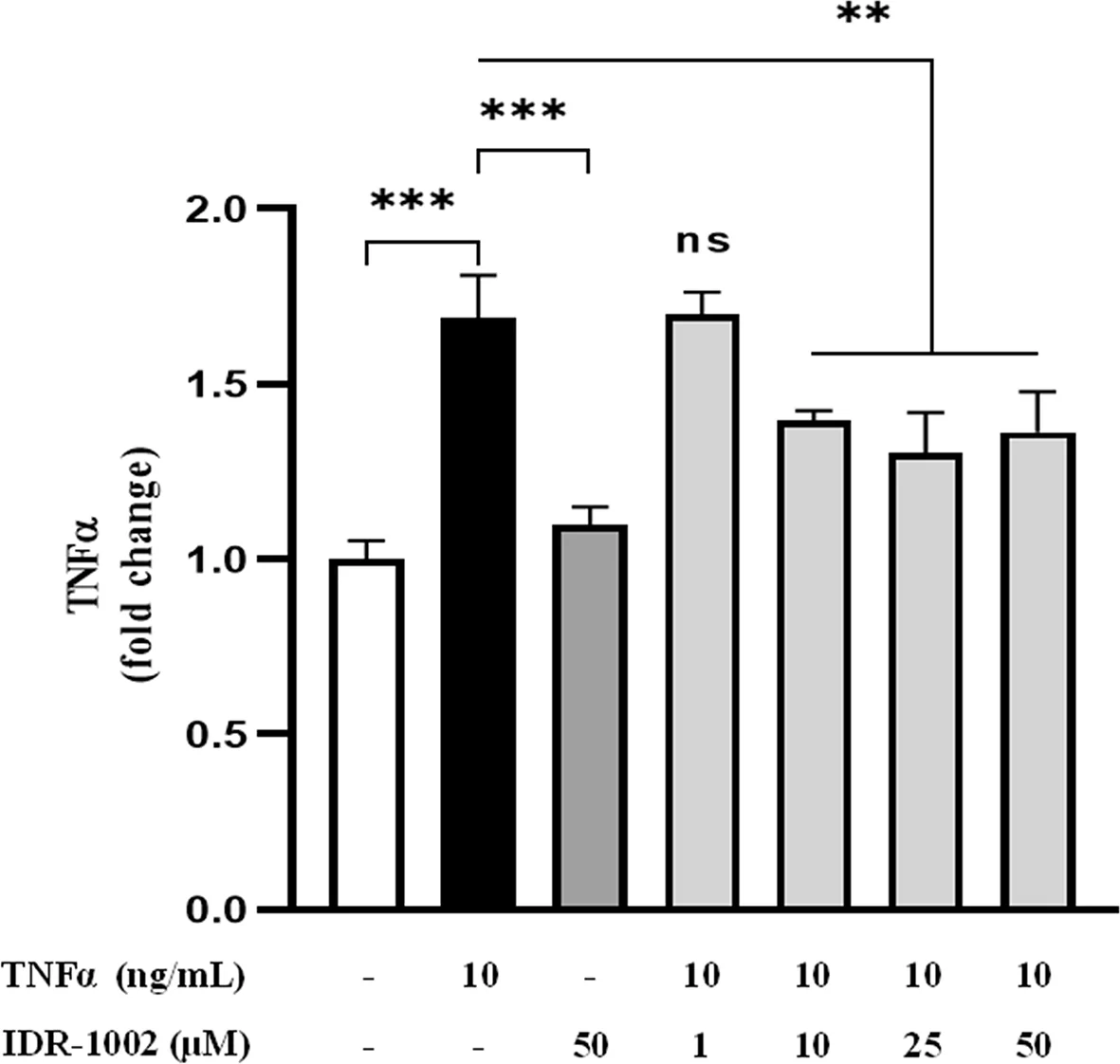
IDR-1002 decreases TNFα expression in BEC cells stimulated with TNFα. BEC cells were pretreated with 1, 10, 25, and 50 μM IDR-1002 for 1 h and subsequently incubated with 10 ng/mL TNFα for an additional 1 h. Supernatants were collected and TNFα levels were quantified by ELISA. These results are representative of three independent experiments (n = 3). Bars indicate the mean ± SD, and asterisks indicate statistical difference, ** p <0.01 ; ***P ˂0.001, n = 3, by the 2-way ANOVA method post hoc Tukey.

To investigate the effect of IDR-1002 on TNFα expression, BEC cells were pre-incubated with 1, 10, 25 and 50 μM IDR-1002 for 1 hour and subsequently incubated with 10 ng/mL TNFα for an additional 1 h. Then, supernatants were collected and TNFα quantification was performed by ELISA. IDR-1002 significantly reduced TNFα levels at concentrations as low as 10 μM ( Figure 5), confirming its anti-inflammatory activity and supporting our previous results as NF-κB inhibitor in macrophage cells. ^21^

## Discussion

This study demonstrates that the immunomodulatory 12-residue cationic peptide IDR-1002 effectively activates the Keap1–Nrf2 signaling pathway, thereby promoting both antioxidant and anti-inflammatory responses in both human and bovine cell models. These findings support the hypothesis that IDR-1002, previously characterized as an NF-κB inhibitor, can also function as a potent activator of Nrf2, highlighting its potential as a dual-function therapeutic agent in conditions associated with oxidative stress and inflammation. The nuclear translocation of Nrf2 induced by IDR-1002 in both HEK293 and BEC cells was dose-dependent and correlated with a significant increase in ARE-luciferase reporter activity in HepG2 cells, with an EC _5__0_ under 20 μM. These results are comparable to those reported for established Nrf2 activators such as TBHP and SFN, albeit IDR-1002 may offer advantages in specificity, null cytotoxicity, and cytocompatibility.

In addition, IDR-1002 upregulated the expression of key phase II antioxidant enzymes including HO-1, NQO1, and GCLM, as well as activation of GST. These enzymes play a central role in maintaining cellular redox homeostasis and mitigating oxidative damage. Of note, GST activity remained elevated up to 24 h post-treatment, suggesting a sustained cellular response and prolonged protective effect. Since GSTs mediate the binding of glutathione to electrophilic compounds, promoting their clearance and reducing the cumulative damage caused by ROS and lipid peroxidation-derived products, ^45^ their sustained activation could provide a global defense against recurrent or prolonged oxidative stress episodes. Consistent with these antioxidant effects, IDR-1002 significantly reduced intracellular ROS levels following H _2_O _2_ stimulation. This attenuation of oxidative stress likely contributes to its anti-inflammatory activity, as evidenced by a marked reduction in TNFα protein levels in TNFα-stimulated BEC cells. These results are in agreement with our previous report demonstrating that IDR-1002 can inhibit NF-κB activation in macrophages by preventing IκBα degradation and p65 nuclear translocation. ^21^ Therefore, IDR-1002 exerts simultaneous modulatory effects on both Nrf2 and NF-κB signaling pathways, positioning it as a promising synthetic immunomodulatory candidate with a dual function.

Based on its reported capacity to inhibit the NF-κB pathway and activate Nrf2, IDR-1002 shares functional similarities with several naturally occurring peptides such as YD1 (a decapeptide from kimchi), ^11^ K-8-K (an octapeptide from milk), and S-10-S (a decapeptide from soy). ^13^ These peptides promote Nrf2 nuclear translocation and suppress inflammation, at least in part, by preserving IκB. Another peptide in this category is LP-5, a pentapeptide derived from walnut protein, that mitigates oxidative stress and inflammation through Nrf2 activation, increasing the activity of superoxide dismutase and catalase, and reducing the activation of the NLRP3 inflammasome. ^14^ While these natural peptides offer promising biological profiles, IDR-1002 provides distinct advantages, including synthetic accessibility, the modularity of its amino acid sequence for optimization, and a well-characterized immunomodulatory profile across diverse models.

The broader immunomodulatory activities previously described for IDR peptides help contextualize the differential Nrf2 responses observed in this study. Evidence from other innate defense regulator peptides supports the dual antioxidant and anti-inflammatory profile observed for IDR-1002. Studies in human neutrophils have shown that IDR-1018 and HH2 reduce ROS production, suppress TNF-α release, and promote LL-37 secretion, demonstrating that IDRs can simultaneously modulate oxidative and inflammatory pathways. ^17^ Additionally, IDR-1 has been reported to interact with the ZZ domain of p62/SQSTM1, a key regulator of the Keap1–Nrf2 axis, providing a mechanistic explanation on how certain IDRs may facilitate Nrf2 stabilization through p62-dependent sequestration of Keap1. ^46^ The minimal Nrf2 activation observed for IDR-1 in our experiments aligns with the notion that distinct IDR sequences differentially engage components of the Nrf2 pathway. Conversely, the strong Nrf2 nuclear accumulation induced by IDR-1002 together with its previously reported NF-κB inhibition supports the idea that IDR-1002 integrates both antioxidant and anti-inflammatory activities more effectively than other synthetic related peptides. Thus, our results position IDR-1002 as the most potent dual-function regulator within this peptide family. Although these findings are promising, further experimental validation is necessary to confirm direct binding between IDR-1002 and its biological target in order to determine if this interaction modulates Keap1-mediated degradation. Based on our previous finding that FKC targets TNFR1, ^44^ and our recent observations that FKC also activates Nrf2, we investigated if IDR-1002 shared this mechanism. However, FRET-based monitoring of TNFR1 conformational dynamics showed no inter-monomeric changes, indicating that, unlike FKC, IDR-1002 is not a direct receptor antagonist or allosteric modulator. Consequently, its dual activity must involve an alternative receptor or signaling node, narrowing the search for its primary cellular target.

Finally, despite the encouraging evidence presented here, a deep insight on the pharmacokinetic properties, bioavailability, and potential off-target effects require further investigation. Furthermore, validation of these findings in animal models of oxidative and inflammatory diseases are mandatory. In spite of these limitations, IDR-1002 emerges as a next-generation of non-cytotoxic dual-function synthetic peptides that simultaneously activates Nrf2 and inhibits NF-κB signaling, which makes it a good therapeutic candidate for diseases involving oxidative stress and inflammation.

## Data Availability

Figshare: Files that contain data on Nrf2 activation by the cationic peptide IDR-1002. https://doi.org/10.6084/m9.figshare.31093330. ^47^
○ WB peptides IDR, densitometry data.xlsx (Data used to generate Figures 1A-D). ○ WB Nrf2 data analysis HEK293 cells; peptides IDR.pzfx (Data used for statistical analysis of Figures 1A and D). ○ Densitometry data WB in BEC cells with (IDR-1002).xlsx (Data used to generate Figures 1E and F; Figures 2B and C). ○ WB Nrf2 data analysis BEC cells; IDR-1002.pzfx (Data used for statistical analysis of Figures 1E and F). ○ HepG2 EC50 assay IDR-1002; raw and normalized data.pzfx (Data used to generate Figure 2A). ○ HO-1_NQO1_GCLM ELISA normalized data 2h and 4h.pzfx (Data used to generate Figures 2E-F). ○ GST activity assay 6,12,18,24 h; raw and normalized data.pzfx (Data used to generate Figure 3). ○ ROS assay; raw data and normalized.pzfx (Data used to generate Figure 4). ○ TNF alpha assay; raw and normalized data.pzfx (Data used to generate Figure 5). Data are available under the terms of the Creative Commons Attribution 4.0 International license (CC-BY 4.0). Figshare: Files that contain the supplementary Figure S1 and data on IDR-1002 cytotoxicity. https://doi.org/10.6084/m9.figshare.31116373.v1. ^48^
○ MTT assay in Hep G2 cells 8 h; raw and normalized data.pzfx (Data used to generate supplementary Figure 1A). ○ MTT assay in bovine endothelial cells (BEC) 8 h; raw and normalized data.pzfx (Data used to generate supplementary Figure 1B). ○ Supplementary FIGURE S1_Romero-Duran-2026.tif Data are available under the terms of the Creative Commons Attribution 4.0 International license (CC-BY 4.0).
