# PEPTIDE IDR-1002 REGULATES THE ANTIOXIDANT AND ANTI-INFLAMMATORY RESPONSES BY ACTIVATING THE KEAP1-NRF2 SIGNALING PATHWAY

**DOI:** 10.12688/f1000research.177148.2

**Published:** 2026-04-28

**Authors:** Marco Antonio Romero-Durán, María Cristina Maldonado-Pichardo, Jose Manuel Perez-Aguilar, Victor Manuel Baizabal Aguirre

**Affiliations:** 1 Centro Multidisciplinario de Estudios en Biotecnología, Facultad de Medicina Veterinaria y Zootecnia, Universidad Michoacana de San Nicolás de Hidalgo, Morelia, Michoacan, 58100, Mexico; 2 Centro Multidisciplinario de Estudios en Biotecnología, Facultad de Medicina Veterinaria y Zootecnia, Universidad Michoana de San Nicolás de Hidalgo, Morelia, Michoacan, 58100, Mexico; 3 School of Chemical Sciences, Meritorious Autonomous University of Puebla, Puebla, Puebla, 72570, Mexico; 4 Centro Multidisciplinario de Estudios en Biotecnología, Facultad de Medicina Veterinaria y Zootecnia, Universidad Michoacana de San Nicolás de Hidalgo, Morelia, Michoacan, 58100, Mexico

**Keywords:** IDR-1002, peptide, Nrf2, oxidative stress, inflammation, TNF-α.

## Abstract

**Background:**

Oxidative stress and inflammation are mutually reinforcing physiological processes. The transcription factor Nrf2 (Nuclear factor erythroid 2-related factor 2) acts as the master regulator of redox homeostasis and anti-inflammatory signaling. To overcome the off-target limitations of conventional electrophilic Nrf2 activators, this study investigates selective peptide-based modulators. We present data indicating that an innate defense regulator (IDR)-1002 peptide is able to activate the Nrf2-mediated antioxidant response and mitigate inflammation in an endothelial cell model.

**Methods:**

We first screened several IDR peptides IDR-1 (Lys-Ser-Arg-Ile-Val-Pro-Ala-Ile-Pro-Val-Ser-Leu-Leu), IDR-1018 (Val-Arg-Leu-Ile-Val-Ala-Val-Arg-Ile-Trp-Arg-Arg), HH2 (Val-Gln-Leu-Arg-Ile-Arg-Val-Ala-Val-Ile-Arg-Ala) and IDR-1002 (Val-Gln-Arg-Trp-Leu-Ile-Val-Trp-Arg-Ile-Arg-Lys) for their ability to induce Nrf2 nuclear translocation in HEK293 and bovine endothelial cells (BEC) via Western blot and ELISA. Transcriptional activity was assessed in HepG2 cells using an ARE (Antioxidant Response Element)-luciferase reporter assay to determine the EC _50_. Downstream expression of antioxidant enzymes, including HO-1 (Heme Oxygenase-1), NQO1 (NAD(P)H:quinone oxidoreductase 1), and GCLM (Glutamate-Cysteine Ligase Modifier Subunit), was quantified by ELISA. GST (Glutathione S-Transferase) activity induced by IDR-1002 was measured using a CDNB-GSH conjugation assay. Finally, the functional capacity to reduce H _2_O _2_-induced ROS (Reactive Oxygen Species) and TNF-stimulated inflammation was measured in BECs.

**Results:**

Among the tested peptides, IDR-1002 induced the most potent concentration-dependent nuclear translocation of Nrf2 in both cell lines. IDR-1002 activated ARE-dependent transcription with an EC _50_ of 18.57 μM. This activation led to a significant time-dependent increase in HO-1, NQO1, and GCLM protein levels, alongside enhanced GST activity. Functionally, IDR-1002 pre-treatment resulted in a dose-dependent reduction of intracellular ROS (up to 5.4-fold at 50 μM) and a significant decrease in TNF-α expression in stimulated BECs.

**Conclusions:**

IDR-1002 acts as a distinct dual-function regulator that simultaneously modulates the Nrf2 antioxidant response and inhibits NF-κB-mediated inflammation. These findings highlight the potential of IDR-1002 as a promising molecular template for the design of new therapeutic approaches against chronic diseases characterized by the interplay between oxidative stress and inflammation.

## Introduction

Reactive oxygen species (ROS) are essential signaling molecules that, at low concentrations, contribute to physiological cellular functions, including cell proliferation, differentiation, and host defense. However, excessive accumulation of ROS or reactive nitrogen species (RNS) disrupts redox homeostasis and induces oxidative stress, leading to damage of lipids, proteins, and DNA that may cause numerous chronic and acute diseases, including neurodegenerative disorders, cancer, cardiovascular diseases, and infections. ^1– 3^

The transcription factor nuclear factor erythroid 2–related factor 2 (Nrf2) is a central regulator of the cellular antioxidant response that orchestrates a protective gene expression program against oxidative and electrophilic stress. ^3– 5^ Under basal conditions, Nrf2 is sequestered in the cytoplasm by the Kelch-like ECH-associated protein 1 (Keap1), which targets it for ubiquitination and proteasomal degradation. Upon exposure to various stress agents, Nrf2 dissociates from Keap1 and translocates to the nucleus, where it binds to the antioxidant response element (ARE) and induces the transcription of phase II detoxification enzymes such as heme oxygenase-1 (HO-1), NAD(P) H quinone oxidoreductase 1 (NQO1), and glutamate–cysteine ligase modifier subunit (GCLM), among others. ^3, 6^

Due to its central role in cytoprotection, Nrf2 is currently a promising therapeutic target for treating diseases associated with oxidative stress and chronic inflammation. Several small-molecule Nrf2 activators, including sulforaphane, chalcone, and fumaric acid derivatives such as dimethyl fumarate (DMF), have been investigated in preclinical and clinical settings. These compounds typically act as electrophiles that modify cysteine residues (e.g., Cys151, Cys273, Cys288) on Keap1 to disrupt its interaction with Nrf2. ^3, 6, 7^ Although these molecules appear to have appropriate characteristics, they have been observed to alter the activity of other proteins bearing Cys residues. This may non-selectively modify other cysteine-containing proteins, leading to off-target effects. Because of these limitations, there is increasing interest in the development of selective peptide-based modulators that can disrupt the Nrf2–Keap1 interaction with higher specificity and fewer side effects. ^8^ Nevertheless, the ability of peptides to simultaneously activate Nrf2 signaling while inhibiting a pro-inflammatory response mediated by nuclear factor kappa B (NF-κB) remains underexplored. ^9– 12^

Interestingly, some natural peptides have shown dual regulatory functions by activating Nrf2 and concurrently inhibiting NF-κB-mediated inflammation. For instance, the decapeptide YD1 (Ala-Pro-Lys-Gly-Val-Gln-Gly-Pro-Asn-Gly) induces an increase in Nrf2 activity and expression of downstream antioxidants such as HO-1 and NQO1, while suppressing pro-inflammatory mediators through TLR4/MyD88/NF-κB pathway inhibition. ^11^ Similarly, peptides such as K-8-K (Lys-Val-Leu-Pro-Val-Pro-Gly-Lys), S-10-S (Ser-Leu-Val-Asn-Asn-Asp-Asp-Arg-Asp-Ser), and LP-5 (Leu-Pro-Val-Thr-Lys), derived from milk, soy, and walnuts, respectively, ^13, 14^ show comparable dual activity by promoting antioxidant enzyme expression and reducing inflammasome activation. These findings highlight that modulation of both oxidative stress and inflammation is an emerging characteristic among certain natural peptides. ^11^

This dual regulatory capacity is particularly relevant for the vascular endothelium. Endothelial cells (ECs) are no longer viewed as passive vascular barriers, but as active immunological sentinels positioned at the critical interface between systemic circulation and tissues. As primary sensors of hemodynamic and chemical stimuli, ECs maintain vascular homeostasis through a unique metabolic profile that relies heavily on glycolysis to minimize mitochondrial ROS production. ^15, 16^ This strategic position allows them to function as early checkpoints in the transition from systemic inflammatory cues to localized tissue injury. ^17^ Upon encountering inflammatory stimuli, ECs rapidly respond by producing pro-inflammatory cytokines, most notably tumor necrosis factor-alpha (TNF-α). This production triggers a cascade that upregulates adhesion molecules (e.g., ICAM-1 and VCAM-1) and chemokines, orchestrating leukocyte recruitment and transmigration into the parenchyma. ^18, 19^ Beyond immune cell trafficking, endothelial-derived TNF-α directly disrupts intercellular tight junctions (e.g., ZO-1 and Occludin), leading to vascular ‘ *leakiness*’ and subsequent tissue damage. ^20^ Therefore, fortifying this “ *vascular gateway*” through the Keap1-Nrf2 signaling pathway represents a strategic approach to mitigating chronic inflammation and oxidative stress. In this study, we utilize Bovine Endothelial Cells (BECs) as a high-fidelity model. BECs provide a robust representation of large-mammal vascular responses and hold significant clinical relevance in bovine-specific inflammatory conditions, such as mastitis and respiratory disease. ^21^ Given the importance of the endothelium in the inflammatory response, synthetic peptides derived from natural host defense templates represent a valuable strategy for targeted intervention.

In this context, the innate defense regulator (IDR) anti-inflammatory peptides IDR-1 (Lys-Ser-Arg-Ile-Val-Pro-Ala-Ile-Pro-Val-Ser-Leu-Leu), ^22, 23^ IDR-1018 (Val-Arg-Leu-Ile-Val-Ala-Val- Arg-Ile-Trp-Arg-Arg), ^17^ HH2 (Val-Gln-Leu-Arg-Ile-Arg-Val-Ala-Val-Ile-Arg-Ala), ^24^ and IDR-1002 (Val- Gln-Arg-Trp-Leu-Ile-Val-Trp-Arg-Ile-Arg-Lys) ^24– 26^ emerge as good candidates to test their ability to modulate the activity of Nrf2.

In particular, IDR-1002 was identified from a library of bactenecin, an antimicrobial peptide found in bovine neutrophils, ^25^ as a peptide able to confer protection against invasive *Staphylococcus aureus* infection through chemokine induction. ^26^ Furthermore, IDR-1002 significantly reduced the production of reactive oxygen and nitrogen species (ROS/RNS) and attenuated inflammation and tissue damage *in vivo* in a mouse ear inflammation model. ^27^ Our group has also previously reported that IDR-1002 modulates inflammation in RAW 264.7 macrophages challenged with lipopolysaccharide (LPS), TNFα, or IL-1β via inhibition of IκBα phosphorylation and NF-κB p65 nuclear translocation. ^28^ Based on these findings, we hypothesized that IDR-1002 may also be able to upregulate an antioxidant response through Nrf2 signaling in addition to its known anti-inflammatory activity. Supporting this notion, another study using a chicken hepatocyte non-parenchymal cell co-culture showed that IDR-1002 reduces pro-inflammatory cytokine release while increasing Nrf2 production, highlighting its dual role in regulating inflammation and antioxidant responses; however, definitive proof of its direct effects on Nrf2 activation and subsequently the downstream antioxidant enzyme expression was not assessed. ^12^

Thus, in this study, we present experimental evidence regarding the potential of IDR-1002 to modulate Nrf2-mediated antioxidant responses in human embryonic kidney (HEK293) cells and bovine endothelial cells (BEC). Our data demonstrate that IDR-1002 induces Nrf2 nuclear translocation, enhances ARE-driven transcriptional activity, upregulates the production of key antioxidant enzymes (HO-1, NQO1 and GCLM), and promotes Glutathione S-Transferase (GST) activity. Furthermore, IDR-1002 reduced general oxidative stress in H _2_O _2_-stimulated cells and attenuated TNF-α production in TNFα-challenged BECs. Together, these results highlight the dual regulatory potential of IDR-1002 on redox and inflammatory pathways under the experimental conditions tested. These findings suggest that IDR-1002 could serve as a promising molecular scaffold for the development of peptide-based agents aimed at fine-tuning the Nrf2 and NF-κB signaling axes.

## Materials and methods

### Peptide synthesis

The immunomodulatory peptides IDR-1018 (VRLIVAVRIWRR-NH2), IDR-HH2 (VQLRIRVAVIRA-NH2), IDR-1 (KSRIVPAI-PVSLL-NH2), and IDR-1002, (VQRWLIVWRIRK-NH2) with the C-terminal amidated were synthesized by solid-phase Fmoc chemistry (CPC Scientific, USA). ^29^ The purity of the synthetic peptides was confirmed to be greater than 95% by high-performance liquid chromatography (HPLC) and mass spectrometry (MS). Lyophilized peptides were reconstituted in sterile water or PBS and stored at −80°C until use.

### Antibodies and reagents

Primary and secondary antibodies used: mouse β-actin (sc-47778), laminin (sc-7293), GAPDH-IgG (sc-32233), and anti-mouse IgG-BP-HRP (sc-525409) (Santa Cruz Biotechnology, USA); Histone H3 Rabbit (9717), rabbit NQO1 (62262S), and anti-rabbit IgG-HRP (7074S) (Cell Signaling Technology, USA); rabbit Nrf2 (ADI-KAP-TF125) and HO-1 (ADI-OSA-150-F) (Enzo Life Sciences, USA). Additional reagents included non-fat dry milk (Bio-Rad, USA), Luminol (Millipore, USA), protease and phosphatase inhibitor cocktail (cOmplete™, Roche, Switzerland), tert-butyl hydroperoxide (TBHP; Sigma-Aldrich, cat. 458139), ferrous sulfate (FeSO _4_; cat. 7782-63-0), D, L-sulforaphane (SFN; cat. S4441), Tris-HCl, NaCl, 1-chloro-2,4-dinitrobenzene (CDNB), hydrogen peroxide (H _2_O _2_; cat. 7722-84-1), DCFDA (cat. 4091-99-0), Igepal CA-930, Na-pyrophosphate, NaF, Na-orthovanadate, RIPA buffer (cat. R0278), and chemiluminescent substrate (Millipore, USA). TNFα was purchased from (ACROBiosystem-Switzerland; cat. TNA-H4211). Cell culture media and supplements were obtained from BPS Bioscience (USA), including MEM, Thaw Medium 1, 1K nutrient medium, fetal bovine serum (FBS), non-essential amino acids, sodium pyruvate, penicillin/streptomycin, geneticin (cat. 79533), and Luciferase ONE-Step reagent (cat. 60690). Trypsin-EDTA 1X (cat. T2601), L-glutathione reduced (gsh, cat. G4251), Bradford Reagent (cat. B6916) and MTT (3-(4,5 dimethylthiazol-2-yl)-2,5-diphenyltetrazolium bromide (cat. 475989) were purchased from Sigma-Aldrich.

### Cell culture

ARE-Hep-G2 cells (Hep-G2 cells) (BPS Bioscience, cat. 60513) which express luciferase under control of ARE sequences were cultured in 1K medium containing DMEM supplemented with 10% FBS, 1% non-essential amino acids, 1 mM sodium pyruvate, 1% penicillin/streptomycin, and 600 μg/mL geneticin at 37°C in a humidified atmosphere with 5% CO _2_. Cells between passages 8–23 were used. BEC cells (bovine umbilical vein endothelial cells) were kindly provided by Dr. Carmen Clapp from the Institute of Neurobiology, National Autonomous University of Mexico (UNAM). These cells were immortalized by transfection with HPV16 E6E7 oncogenes, that extend their replicative lifespan of primary BEC from 40 to more than 120 passages without signs of senescence. Importantly, BEC cells retain key endothelial characteristics, including the uptake of acetylated low-density lipoproteins, von Willebrand factor expression, specific lectin binding, and proliferative responses to vascular endothelial growth factor. ^21^ HEK-293 cells were purchased from the American Type Culture Collection (ATCC; cat. CRL-1573). HEK-293 and BEC cells were cultured in DMEM supplemented with 10% FBS, 10,000 U/mL penicillin, and 1 mg/mL streptomycin, at 37°C in 5% CO _2_. Cells between passages 8 and 20 were used.

### Luciferase reporter assay

Hep-G2 cells ^30^ were seeded at a density of 40,000 cells/well in 96-well plates using 45 μL of 1 K medium without geneticin. Cells were treated with 5 μL of IDR-1002 (final concentration: 0.5–300 μM) or FeSO _4_ (100 μM) as a positive control. Following an 8–10 h incubation period at 37°C, 100 μL of Luciferase ONE-Step reagent was added, and plates were shaken for 15 min. Luminescence was measured using a Varioskan ^TM^ LUX multimode microplate reader (Thermo Fisher Scientific, USA). To ensure high signal-to-noise ratio and data accuracy background signal and cellular autofluorescence were subtracted from all treatment groups. Fold induction was calculated by normalizing the corrected luminescence values to the untreated controls. Tert-butyl hydroperoxide (TBHP), FeSO _4_, and sulforaphane (SFN) were utilized as established Nrf2-pathway activators.

### MTT cell viability assay

Hep-G2 and BEC cells were seeded in 96-well plates at 5 × 10 ^4^ cells/mL and incubated for 24 h at 37°C. Cells were then treated with IDR-1002 (1–100 μM) or TBHP (10 μM) for 8 h. Following treatment, 50 μL of MTT solution (5 mg/mL in PBS, for a final concentration of 10% v/v) was added to each well and incubated for 4 h at 37°C. The culture medium was then carefully aspirated, and the resulting formazan crystals were solubilized in 100 μL of 100% DMSO. Absorbance was read at 570 nm using a VarioskanTM LUX reader (Thermo Fisher Scientific, USA). Cell viability was calculated as a percentage relative to the untreated control cells (100% viability), and background absorbance from cell-free blank wells was subtracted from all readings to ensure data accuracy.

### Assessment of general intracellular oxidative stress

General intracellular oxidative stress levels were determined using 2′,7′-dichlorodihydrofluorescein diacetate (DCFH-DA; Sigma-Aldrich, USA). ^31– 33^ BEC cells were seeded in black-walled, clear-bottom 96-well plates (Corning, USA) to prevent fluorescence cross-talk between adjacent wells. Cells were pretreated with IDR-1002 (1–50 μM) for 4 h and subsequently incubated with 30 μM DCFH-DA in PBS at 37°C for 30 min in the dark. During this period, the non-fluorescent DCFH-DA is internalized and hydrolyzed by intracellular esterases into DCFH. Following incubation, cells were washed with PBS to remove the extracellular probe and then stimulated with 50 μM H _2_O _2_ for 20 min. The oxidation-mediated conversion of DCFH to the highly fluorescent 2′,7′-dichlorofluorescein (DCF) was quantified using a Varioskan ^TM^ LUX multimode microplate reader (Thermo Fisher Scientific, USA) at excitation/emission wavelengths of 485/530 nm. To ensure data accuracy, background fluorescence was corrected by subtracting the signal from cell-free blank wells (to account for probe auto-oxidation) and untreated control cells (to account for cellular autofluorescence). Results were expressed as Relative Intracellular Redox State (Fold Change) normalized to control groups.

### Protein extraction and Western blotting

BEC cells were grown in 6-well plates to ~90% confluence, serum-starved for ≥4 h, and then treated with 1, 10, 25, or 50 μM IDR-1002 for 1 h or 4 h before lysis for subsequent Western blots of Nrf2, HO-1, and NQO1. Total protein (cytosolic plus nuclear from control and treated cells) was extracted by washing cells with cold PBS and lysing them with 80 μL of a cold buffer containing 20 mM Tris–HCl pH 7.5, 150 mM NaCl, 1% Igepal CA-930, 10 mM Na-pyrophosphate, and 50 mM NaF supplemented with 1 mM Na-orthovanadate and 1x protease inhibitors. Lysates were centrifuged at 13,000 × g (20 min, 4°C), and supernatants collected and transferred to ice-cold Eppendorf tubes. For subcellular distribution studies, cytoplasmic and nuclear fractions were isolated using the NE-PER Nuclear and Cytoplasmic Extraction Kit (Thermo Scientific) according to the manufacturer’s instructions. The resulting nuclear pellets were resuspended in 80 μL of RIPA buffer and sonicated with a Qsonica Q125 sonicator (Newtown, USA) at 25% amplitude for 15 s (3 × 5 s cycles, with 7 s rest intervals) to ensure complete protein solubilization. ^28^ Protein concentration was measured by the Bradford method using BSA as standard. ^34^ Samples (50-60 μg) were separated by 10% SDS-PAGE and transferred in a wet chamber to 0.22 μm nitrocellulose membranes for 1 h at 250–300 mA. Detection was performed using appropriate antibodies and Immobilon HRP chemiluminescent kit. Imaging was done with the LI-COR Odyssey system.

### Nrf2 and antioxidant enzymes quantification

Intracellular levels of Nrf2, HO-1, NQO1, and GCLM were quantified using competitive ELISA kits (MyBioSource, USA). Briefly, 100 μL of standards or cell lysates were added to pre-coated plates along with 10 μL of Balance Solution. Samples were then incubated with 50 μL of HRP-conjugate for 1 h at 37°C, where endogenous antigens competed for conjugate binding sites. After five washes, TMB substrate was added for color development and incubated in the dark at 37°C. The reaction was stopped with 50 μL of Stop Solution and the absorbance was measured at 450 nm. Results were inversely proportional to the target protein concentration and were normalized to total protein content for each sample.

### Glutathione S-transferase activity assay

BEC cells were treated with 50 μM IDR-1002 for 6, 12, 18, or 24 h. Following treatment, cell lysates were prepared in 50 mM Tris-HCl buffer (pH 7.4) containing 150 mM NaCl and protease inhibitors. Total GST activity was determined by monitoring the conjugation of 1-chloro-2,4-dinitrobezene (CDNB) with reduced glutathione (GSH). Briefly, cell lysates (containing 35 μg of total protein) were incubated with a reaction mixture consisting of 1 mM CDNB and 1 mM GSH (prepared from 100 mM concentrated stocks) in a phosphate-buffered saline (PBS) solution adjusted to pH 6.5. The enzymatic reaction was monitored at 340 nm using a microplate reader every 30 seconds for 5 minutes at [30°C] to determine the initial reaction velocity ( *V*_0_). ^35– 37^

To account for non-enzymatic (spontaneous) conjugation of CDNB, a reagent blank (without cell lysate) was included in each run. This background absorbance was subtracted from all experimental readings to calculate the net enzymatic activity. Specific activity was determined using the molar extinction coefficient for the GSH-CDNB conjugate ( *ε* = 9.6 mM ^−1^cm ^−1^) and a light path of [0.6 cm for a 96-well plate or 1 cm for a cuvette]. Final values were calculated as units of activity per milligram of protein (U/mg). To account for variations in basal enzymatic levels across independent experiments and facilitate the comparison of biological induction, data were normalized and expressed as fold change relative to the unstimulated control (CTRL).

### TNFα quantification

Human TNFα levels in BEC supernatants were quantified by a sandwich enzyme-linked immunosorbent assay (ELISA) using the MBS267654 kit (MyBioSource, USA). Briefly, cell culture supernatants were centrifuged at 1000–3000 rpm for 10 min to remove debris. Standards (ranging from 15.6 to 1000 pg/mL) and 100 μL of samples were added to pre-coated wells and incubated at 37°C for 90 min. Following two washes with 350 μL of buffer, 100 μL of biotinylated detection antibody (1:100) was added and incubated at 37°C for 60 min. After three additional washes, the plate was incubated with 100 μL of HRP-avidin conjugate for 30 min at 37°C. The colorimetric reaction was developed by adding 100 μL of TMB substrate for up to 30 min in the dark at 37°C, then stopped with 100 μL of Color Reagent C (H _2_SO _4_). Absorbance was measured at 450 nm using a microplate reader within 10 minutes of stopping the reaction. Sample concentrations were determined by interpolation from the standard curve, with a minimum detectable sensitivity of 5 pg/mL.

### Statistical analysis

All data are presented as mean ± standard deviation (SD). Analyses were conducted using GraphPad Prism 8 (GraphPad Software, USA). Unpaired Student’s t test was used for two-group comparisons while one-way ANOVA with Tukey’s post hoc test was applied for multiple comparisons. Statistical significance is indicated by * *P* < 0.05, ** *P* < 0.01, *** *P* < 0.001, **** *P* < 0.0001; ns = not significant and ND = not detected. We employed Tukey’s post-hoc test as it provides a rigorous framework for all-to-all comparisons while strictly controlling the family-wise error rate, ensuring that the observed dose-dependent effects of IDR-1002 are statistically robust across all experimental groups as established in the Honestly Significant Difference (HSD) methodology. ^38^

## Results

### IDR peptides promote Nrf2 nuclear translocation in HEK293 cells

Several synthetic protein-protein interaction (PPI) inhibitors of Keap1-Nrf2 have been reported to activate the Nrf2 pathway; however, their use is limited by low selectivity and cytotoxicity effects. ^39, 40^ In this context, IDR peptides have emerged as promising alternatives due to their immunomodulatory properties and low cytotoxic potential. ^24, 41– 43^ Notably, among the IDR peptides group, IDR-1002 exhibits a potent inhibitory NF-κB activity. ^1,^ ^28^ Thus, we initially explored the ability of IDR-1002 and three other IDR peptides, namely IDR-1018, IDR-HH2, and IDR-1, to activate Nrf2 nuclear translocation. HEK293 cells were selected as the initial screening model due to their robust metabolic machinery and validated resistance to oxidative stress, ensuring a reliable platform for studying signal transduction pathways. ^44^

**Figure 1.**
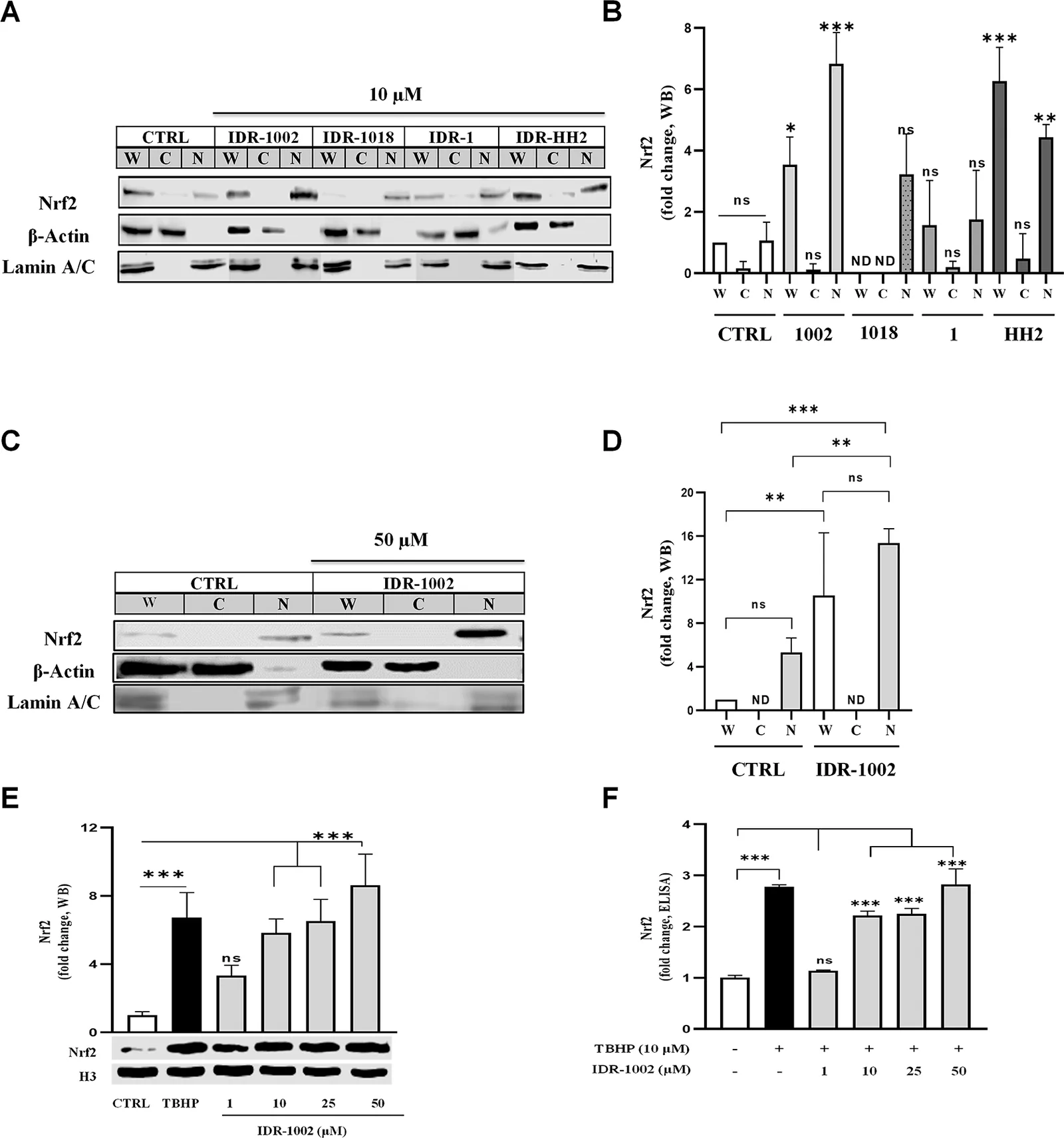
Nrf2 nuclear translocation induced by IDR peptides in HEK293 cells and by IDR-1002 in bovine endothelial cells (BEC cells). ( **A, C**) Western blot detection showing the relative abundance of the Nrf2 protein (MW ~100 kDa) in whole (W), cytoplasmic (C), and nuclear (N) protein enriched extracts from HEK293 cells. Cells were treated for 1 h with 10 μM IDR-1002, IDR-1018, IDR-1, and IDR-HH2 or with 50 μM IDR-1002. β-Actin (MW ~42 kDa) and laminin (MW ~70 kDa) were used as loading controls for the cytoplasmic and nuclear extracts, respectively. **(B, D)** Nrf2 nuclear accumulation is expressed as fold changes normalized to the untreated control (CTRL). **(E)** Representative Western blot showing nuclear Nrf2 levels in BEC cells treated for 1 h with 1, 10, 25, and 50 μM IDR-1002 and a positive control, tert-butyl hydroperoxide (TBHP, 10 μM). The graph above the blot shows the densitometric analysis of the relative fold change in nuclear Nrf2 levels, normalized to the untreated control (CTRL). Histone H3 (H3, 17 kDa) was used as a nuclear loading control. **(F)** Quantification of nuclear Nrf2 levels by competitive ELISA in BEC cells treated under the same conditions as in (E). Mean values for IDR-1002 at 10, 25, and 50 μM, as well as TBHP, were significantly increased compared to CTRL. Comparisons not indicated by asterisks were not statistically significant (ns) or not detect (ND). These results are representative of three independent experiments (n = 3). Bars indicate the mean ± standard deviation (SD). Asterisks indicate a statistically significant difference, determined by two-way ANOVA, followed by Tukey's post hoc test with the following significance levels: *p < 0.05, **p < 0.01 and ***p < 0.001.

HEK293 cells were treated with 10 μM of each peptide and Nrf2 abundance was assessed in whole-cell, cytoplasmic, and nuclear fractions by Western blot ( Figure 1A, B). Among all peptides tested, IDR-1002 induced the strongest nuclear translocation of Nrf2. IDR-HH2 also promoted Nrf2 nuclear accumulation, albeit to a lesser extent, while IDR-1 had minimal effect. To confirm the dose-dependency of IDR-1002, increasing concentrations (up to 50 μM) were tested ( Figure 1C, D). A 15-fold increase in nuclear Nrf2 abundance was observed at 50 μM compared to a 6-fold increase at 10 μM, supporting IDR-1002 as the most potent Nrf2 activator among the tested peptides. These data suggest that sequence variations between IDR peptides influence their ability to modulate the Nrf2 signaling pathway. Based on these results and those we previously reported on NF-κB inhibition, ^28^ IDR-1002 was selected for subsequent experiments.

### IDR-1002 activates Nrf2 nuclear translocation in BEC cells

After identifying IDR-1002 as a lead candidate for Nrf2 modulation in the robust HEK293 signaling model, ^44^ our objective was to determine whether this effect was conserved in a physiologically specialized context. Given the central role of endothelial cells in redox signaling and inflammation, and the antagonistic interplay between NF-κB and Nrf2 in vascular homeostasis, ^26^ ^,^ ^45^ ^–^ ^48^ we chose BEC cells as a relevant model for evaluating peptide-driven cytoprotection. As shown in ( Figure 1E), treatment with increasing concentrations of IDR-1002 induced a pronounced, concentration-dependent increase in Nrf2 nuclear translocation in BECs. These findings were further corroborated by ELISA quantification of Nrf2 levels in BEC lysates, which showed a significant increase following treatment with the peptide from 10 to 50 μM ( Figure 1F). Collectively, these data indicate that IDR-1002 effectively activates the Nrf2 nuclear translocation in both HEK293 and BEC cells, reinforcing the peptide’s potential to fortify the vascular gateway against oxidative and inflammatory challenges.

### IDR-1002 induces Nrf2 transcriptional activity

Under basal conditions, Nrf2 is retained in the cytoplasm by the kelch domain of Keap1. Upon activation, Nrf2 dissociates from Keap1, translocates to the nucleus and binds to ARE in target genes promoters, driving the expression of antioxidant genes. ^49– 51^

**Figure 2.**
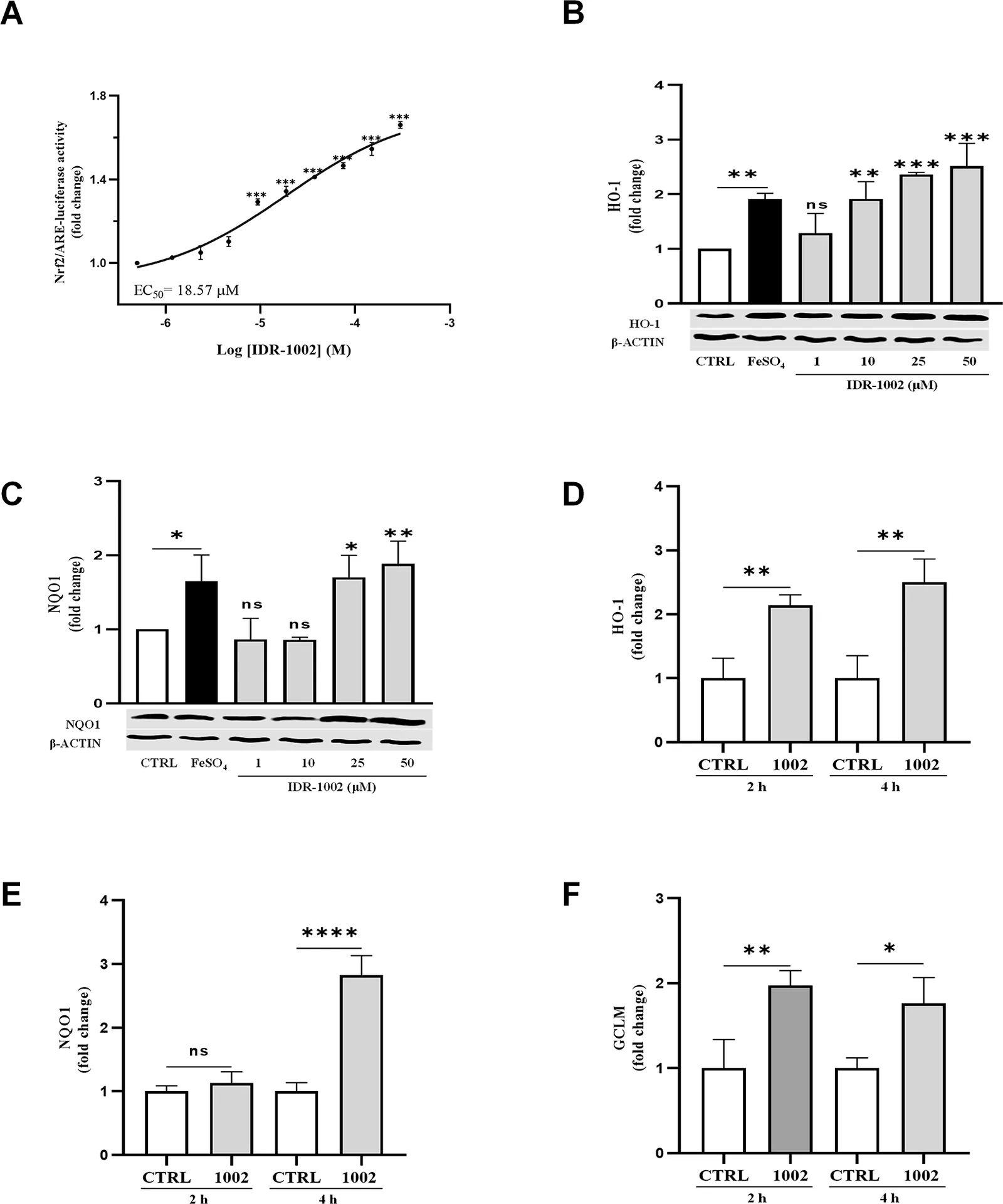
IDR-1002 activates the Nrf2 transcriptional pathway and downstream phase II antioxidant enzymes in BEC cells. **(A)** Measurement of Nrf2-mediated transcriptional activity in HepG2-ARE-luciferase reporter cells treated with IDR-1002 (0.5 to 300 μM) for 8 h. Activity is measured as a fold change in luminescence relative to the untreated control, resulting in an EC _50_ of 18.57 μM. **(B, C)** Western blot analysis of the protein levels of the antioxidant enzymes HO-1 (28 kDa) and NQO1 (29 kDa) in BEC cells treated for 4 h with 1, 10, 25, and 50 μM of IDR-1002. β-Actin (45 kDa) served as a loading control for both blots. Iron (II) sulfate (FeSO _4_, 150 μM) was used as a positive control. Iron (II) sulfate (FeSO _4_, 150 μM) was used as a positive control. The graph above the blot represents the densitometric analysis of the relative fold change in HO-1 expression, normalized to the unstimulated control. Mean values for HO-1 at 10, 25, and 50 μM IDR-1002, and those for NQO1 at 25 and 50 μM IDR-1002 were significantly different from the control group. **(D, E, F)** ELISA quantification of the protein levels of the antioxidant enzymes, HO-1 **(D),** NQO1 **(E),** and GCLM **(F)** in BEC cells treated with 50 μM of IDR-1002 for 2 or 4 h. Protein levels were normalized to total protein content and compared to the respective time-matched untreated control (CTRL 2 h or CTRL 4 h). Treatment with IDR-1002 significantly increased the production of these antioxidant enzymes in a sustained manner across the evaluated time points. Comparisons not indicated by asterisks were not statistically significant (ns) or not detect (ND). These results are representative of three independent experiments (n = 3). Bars indicate the mean ± standard deviation (SD). Asterisks indicate a statistically significant difference, determined by two-way ANOVA, followed by Tukey’s post hoc test with the following significance levels: *p < 0.05, **p < 0.01 and *** *p* < 0.001.

To further explore the cytoprotective potential of IDR-1002 in a tissue condition characterized by high oxidative load and detoxification demands, we evaluated Nrf2 transcriptional activity in HepG2 liver cells stably expressing an ARE-luciferase reporter. This cell line is a well-established gold standard for quantifying the potency of Nrf2-ARE signaling, as it acts as a master regulatory sensor that coordinates redox homeostasis. ^52^ Following stimulation of HepG2 cells with IDR-1002 for 8 h, a concentration-dependent increase in ARE-luciferase activity was observed with an EC _50_ of 18.57 μM ( Figure 2A). Importantly, cell viability assays confirmed that IDR-1002 did not induce cytotoxicity neither in Hep-G2 or BEC cells at 1, 10, 25, 50, or 100 μM ( **Extended data Figure E1**).

### IDR-1002 induces the production of antioxidant enzymes

The Nrf2-dependent phase II antioxidant enzymes HO-1, NQO-1 and GCLM mitigate oxidative stress and can suppress NF-κB activity. ^7^ ^,^ ^51^ ^,^ ^53^ ^,^ ^54^ Western blot analysis confirmed the increased production of HO-1 and NQO1 following IDR-1002 treatment ( Figure 2B, C), supporting its role in Nrf2 activation and downstream antioxidant induction. This was further corroborated by ELISA-based quantification of HO-1, NQO1, and GCLM at 2 and 4 h post-treatment with 50 μM IDR-1002. Treatment with IDR-1002 triggered a significant and sustained upregulation of these antioxidant enzymes. Although the production levels remained elevated from 2 to 4 hours post-treatment, no statistically significant difference was observed between these two time points ( Figure 2D, E, F). This indicates that the induction reaches a plateau or remains stable following the initial response, maintaining the antioxidant defense throughout the evaluated period.

### IDR-1002 induces glutathione S-transferase activity

**Figure 3.**
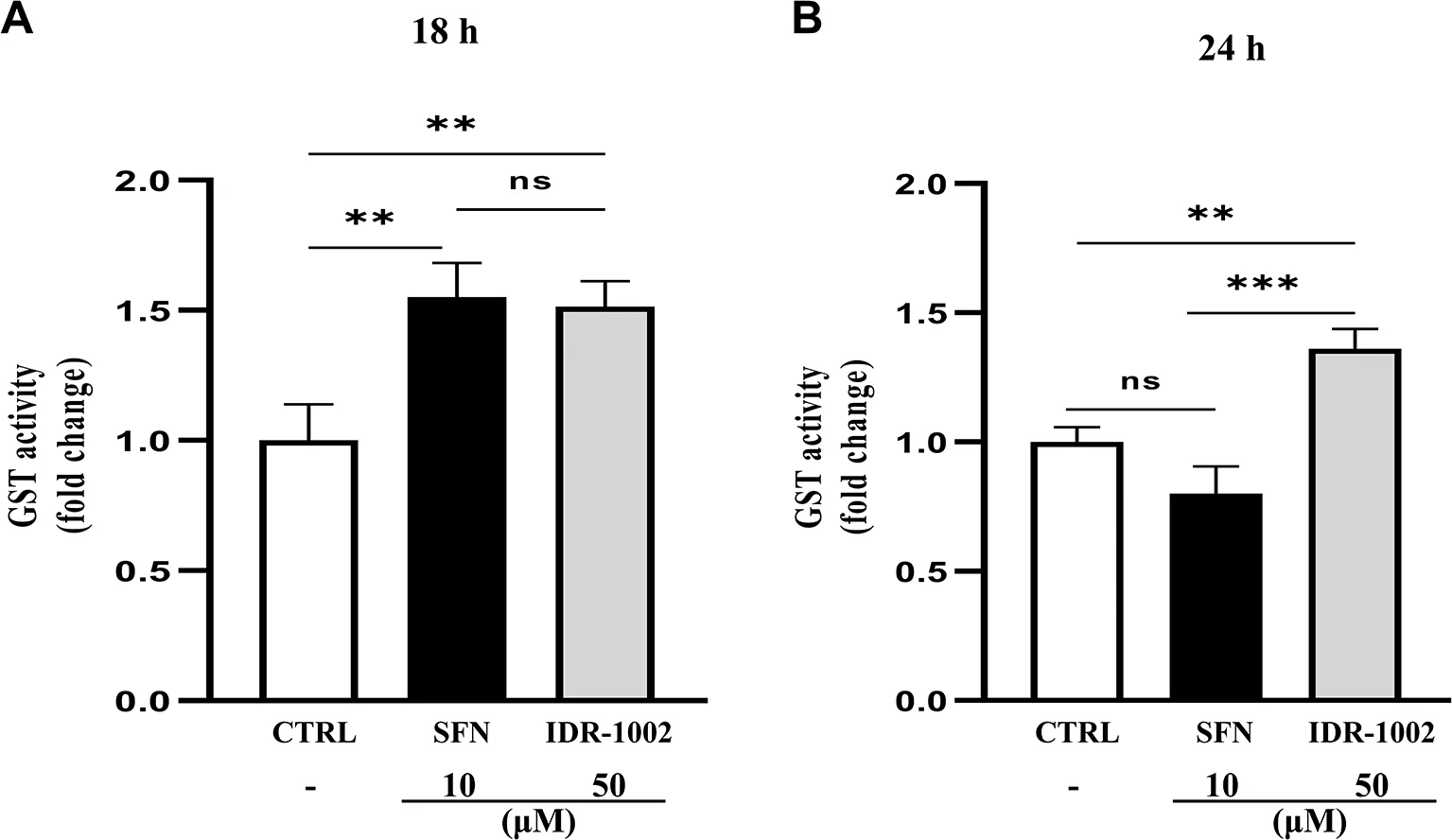
Induction of GST activity by IDR-1002 in BEC cells. BEC cells were treated with 50 μM IDR-1002 for 18 ( **A**) and 24 h ( **B**). Sulforaphane (SFN, 10 μM) was used as a positive control. Glutathione S-transferase (GST) activity was quantified after each treatment using the 1-chloro-2,4-dinitrobenzene (CDNB) colorimetric assay. IDR-1002-stimulated cells showed a significant increase in GST activity compared to the untreated control. Data are representative of three independent experiments (n = 3) and are presented as the mean ± standard deviation (SD). Statistical significance was determined by the two-way ANOVA with Tukey’s post hoc test. Asterisks indicate significance levels as follows: *p < 0.05, **p < 0.01, and ***p < 0.001.

Glutathione S-transferases (GSTs) are a family of phase II detoxifying enzymes that catalyze the conjugation of glutathione (GSH) to reactive intermediates, thereby protecting the cell against oxidative damage. ^53^ ^,^ ^55^ To evaluate whether IDR-1002 modulates GST activity, BEC cells were treated with 50 μM of the peptide for 18 and 24 h. GST activity was significantly increased at 18 h ( Figure 3A). At 24 h ( Figure 3B), activity remained elevated relative to the untreated control, although a slight reduction compared to 18 h was observed, without a statistically significant difference between these two time points. No detectable GST activity was observed at earlier time points (6 and 12 h; data not shown). These findings suggest that GST activation occurs at later time points relative to the early induction of HO-1 and NQO1, supporting a temporally distinct response in which Phase II detoxification capacity develops after the initial activation of antioxidant signaling pathways.

### IDR-1002 modulates the intracellular redox state and mitigates general oxidative stress in H _2_O _2_-stimulated BEC cells

**Figure 4.**
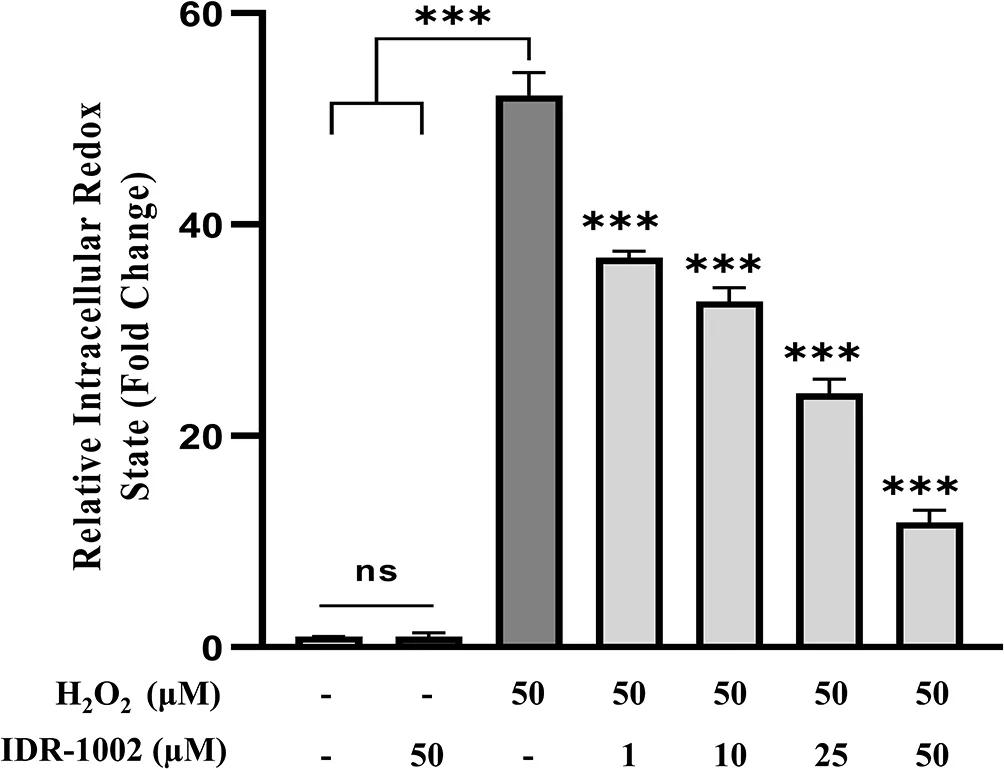
IDR-1002 modulates the intracellular redox state and mitigates general oxidative stress in H _2_O _2_-stimulated BEC cells. BEC cells were pretreated with 1, 10, 25, and 50 μM IDR-1002 for 4 h and subsequently incubated with 50 μM H _2_O _2_ for 15 min to induce oxidative stress. Intracellular ROS production was then quantified using the 2',7'-dichlorofluorescin diacetate (DCFH-DA) fluorescence assay. Data are representative of three independent experiments (n = 3) and are presented as the mean ± standard deviation (SD). Asterisks indicate a statistical difference compared to the H _2_O _2_-treated control, with a significance level of ***p < 0.001.

To assess the antioxidant capacity of IDR-1002, general intracellular oxidative stress was measured in BEC cells challenged with H _2_O _2_ following peptide pretreatment. BEC cells were pre-incubated with the indicated concentrations of IDR-1002 for 4 h and subsequently exposed to 50 μM H _2_O _2_ for 15 min. Basal redox levels remained unchanged in both control and 50 μM IDR-1002-treated cells, indicating that the peptide does not exert pro-oxidant effects under resting conditions ( Figure 4). In H _2_O _2_-stimulated cells, IDR-1002 pre-treatment resulted in a dose-dependent reduction of oxidative stress levels. At 25 and 50 μM, IDR-1002 decreased the fluorescent signal by approximately 2.4- and 5.4-fold, respectively, compared to the H _2_O _2_-only group. These findings are consistent with previous reports in which 18 μM IDR-1002 reduced H _2_O _2_-induced oxidative stress by 1.6-fold in different models. ^12^ These results demonstrate that IDR-1002 effectively modulates the intracellular redox environment most likely through the activation of the Nrf2-mediated antioxidant response.

### IDR-1002 reduces TNF-α production

**Figure 5.**
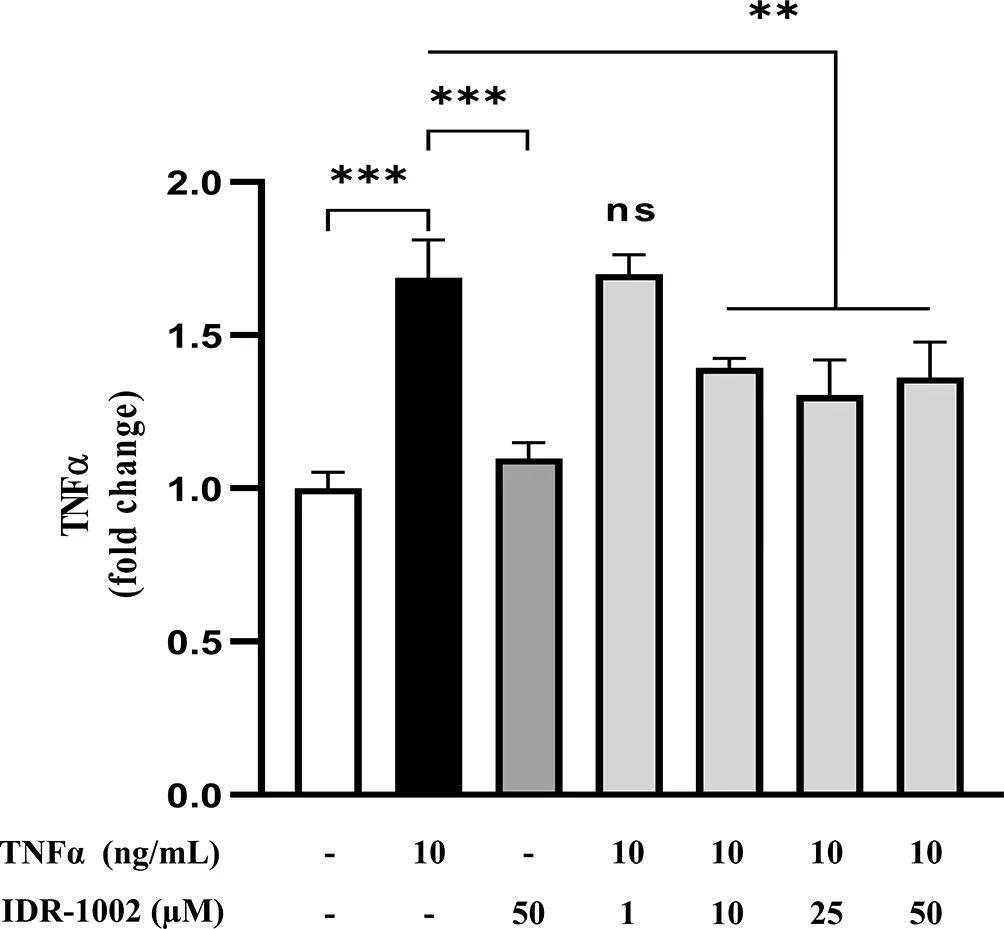
IDR-1002 decreases TNF-α production in BEC cells stimulated with TNFα. BEC cells were pretreated with 1, 10, 25, and 50 μM IDR-1002 for 1 h and subsequently incubated with 10 ng/mL TNFα for an additional 1 h. Supernatants were collected and TNFα levels were quantified by ELISA. These results are representative of three independent experiments (n = 3). Bars indicate the mean ± SD, and asterisks indicate statistical difference, ** p <0.01 ; ***P ˂0.001, n = 3, by the 2-way ANOVA method post hoc Tukey.

Chronic oxidative stress is tightly linked to inflammatory processes, with TNF-α acting as a central mediator in multiple redox-associated pathologies, including cancer, neurodegenerative disorders, metabolic diseases, and cardiovascular conditions. ^56^ ^–^ ^60^ A bidirectional relationship exists between ROS and TNF-α, where oxidative stress can stimulate TNF-α production, which in turn further amplifies intracellular ROS levels, reinforcing the inflammatory response. To evaluate the effect of IDR-1002 on TNF-α production, BEC cells were pre-incubated with 1, 10, 25, and 50 μM of the peptide for 1 h, followed by stimulation with 10 ng/mL TNF-α for an additional 1 h. Supernatants were then collected, and TNF-α levels were quantified by ELISA. IDR-1002 significantly reduced TNF-α production at concentrations ≥10 μM ( Figure 5), supporting its anti-inflammatory activity. These findings are consistent with previous reports describing IDR-1002 as a modulator of NF-κB signaling in macrophages. ^28^

## Discussion

This study demonstrates that the immunomodulatory 12-residue cationic peptide IDR-1002 effectively activates the Keap1–Nrf2 signaling pathway, thereby promoting both antioxidant and anti-inflammatory responses in both human and bovine cell models. The choice of HEK293 cells for the initial characterization of IDR-1002 is supported by recent metabolic profiling, which identifies this lineage as a robust model for studying oxidative stress resistance. According to Sapeta-Nowińska et al. (2025), ^44^ HEK293 cells exhibit a highly efficient adaptive metabolic response, characterized by the precise regulation of key metabolites that support cellular defense mechanisms. This innate resilience makes them an ideal *‘baseline’* to evaluate the efficacy of Nrf2 activators, ensuring that the observed nuclear translocation and subsequent enzymatic induction are mediated by specific signaling modulations rather than generalized cellular distress. Building upon this mechanistic foundation, the inclusion of HepG2 cells in our study strengthens the rationale for IDR-1002 as a systemic modulator of the antioxidant response. While HEK293 cells served as a robust initial model for signaling fidelity, ^44^and BECs offered a context to link Nrf2 activation with its theoretical role in preserving vascular integrity, ^16^ the results in HepG2 cells highlight its potential in high-demand metabolic tissues. The liver is the primary site for neutralizing reactive species and maintaining systemic redox balance, ^52^ consequently, the ability of IDR-1002 to upregulate the Nrf2-driven program in this model suggests a broad therapeutic utility. By activating Nrf2 in HepG2, IDR-1002 demonstrates a capacity to upregulate cytoprotective pathways where oxidative load is prevalent, which suggests a role in mitigating localized vascular damage and systemic oxidative stress through Nrf2-mediated defenses. The inclusion of HEK-293 and HepG2 models alongside BECs provides a comprehensive assessment of IDR-1002’s versatility. By demonstrating consistent activation of the Keap1-Nrf2 pathway across vascular, renal, and hepatic lineages, we establish that the peptide’s anti-inflammatory and antioxidant regulatory effects are part of a conserved, robust pharmacological mechanism and not a cell-specific artifact.

In addition, our data demonstrate that IDR-1002 promotes the induction of Nrf2-dependent proteins, which play a central role in maintaining cellular redox homeostasis and mitigating oxidative damage. The increased production of HO-1, NQO1, and GCLM was significant compared to control groups at both 2 and 4 hours, reflecting a durable activation of the antioxidant response rather than a linear time-dependent increase within the studied temporal window. This sustained profile suggests that IDR-1002 effectively primes the cellular defense mechanism potentially enhancing the cell’s readiness to withstand subsequent oxidative insults. Furthermore, GST activity evaluation suggests that IDR-1002 induces a robust antioxidant response that eventually stabilizes over prolonged periods. Since GSTs mediate the conjugation of glutathione to electrophilic compounds promoting their clearance and reducing cumulative damage from oxidative stress and lipid peroxidation-derived products, ^61^ their significant activity at 24 h strengthens the cellular defense against sustained oxidative challenges. This temporal profile, following the earlier production of HO-1 and NQO1, highlights a coordinated and sequential enzymatic response triggered by the peptide.

Nuclear translocation of Nrf2 induced by IDR-1002 in both HEK293 and BEC cells was dose-dependent and correlated well with a significant increase in ARE-luciferase reporter activity in HepG2 cells, with an *EC*_50_ under 20 μM. These results are comparable in terms of efficacy to those reported for established Nrf2 activators such as TBHP and SFN. ^62^ While SFN exhibits higher potency achieving pathway activation at lower concentrations, IDR-1002 demonstrates a similar maximal induction of the Nrf2-ARE signaling.

Consistent with these antioxidant effects, IDR-1002 significantly reduced intracellular oxidative stress following H _2_O _2_ stimulation. This attenuation of cellular redox imbalance contributes to its anti-inflammatory activity, as evidenced by a marked reduction in TNF- α protein levels in TNF-α-stimulated BECs. The observed decrease in endothelial TNF-α highlights a key anti-inflammatory mechanism operating at the vascular interface. Given that endothelial-derived TNF-α amplifies inflammatory signaling through both autocrine and paracrine pathways, its suppression suggests that IDR-1002 interferes with early events required for leukocyte recruitment and endothelial activation. ^15^ ^,^ ^19^ Of note, TNF-α is also a major driver of endothelial barrier dysfunction, promoting cytoskeletal rearrangement and disruption of tight junction proteins, which leads to increased vascular permeability and tissue injury. ^20^ Elevated endothelial TNF-α is a hallmark of multiple inflammatory pathologies and contributes directly to vascular hyperpermeability and tissue damage. ^17^ Therefore, the ability of IDR-1002 to attenuate TNF-α expression may preserve vascular integrity, limiting both leukocyte recruitment and the propagation of inflammation from the circulation into target tissues. Mechanistically, this effect is in agreement with the established crosstalk between the Nrf2 and NF-κB pathways, whereby activation of Nrf2 negatively regulates NF-κB-dependent transcription of pro-inflammatory mediators, including TNF-α. ^63^ ^,^ ^64^ Hence, the inhibition of TNF-α serves as a functional validation of Nrf2 activation by IDR-1002 that leads to a biologically meaningful anti-inflammatory outcome in a primary endothelial model.

In light of the evidence presented in our previous work, ^28^ regarding the inhibitory effect of IDR-1002 on the IKK/IκBα axis and the activation of p38/ERK1/2–MSK1-dependent CREB phosphorylation, a direct modulation of NF-κB signaling was initially considered. However, the strong association observed here between Nrf2 activation and the induction of Phase II enzymes (HO-1, NQO1, GCLM) supports a more integrated mechanism. Accordingly, we have refined our interpretation to propose the Nrf2 signaling as the primary driver of the observed immunomodulatory phenotype. This model moves beyond a strictly p65-centered explanation and instead supports a mechanism of “ *redox interference*,” which means IDR-1002 promotes a redox-stable intracellular environment through Nrf2 that indirectly antagonizes the pro-inflammatory signaling. ^65^ ^,^ ^66^ By simultaneously preventing IκBα degradation and enhancing antioxidant defenses, IDR-1002 orchestrates a comprehensive cytoprotective response, positioning it as a sophisticated candidate for modulating inflammatory diseases through dual signaling pathways.

Based on its reported capacity to inhibit the NF-κB pathway and activate Nrf2, IDR-1002 shares functional similarities with several naturally occurring peptides such as YD1 (a decapeptide from kimchi), ^11^ K-8-K (an octapeptide from milk), and S-10-S (a decapeptide from soy). ^13^ These peptides promote Nrf2 nuclear translocation and suppress inflammation, at least in part, by preserving IκB. Another peptide in this category is LP-5, a pentapeptide derived from walnut protein, that mitigates oxidative stress and inflammation through Nrf2 activation, increasing the activity of superoxide dismutase and catalase, and reducing the activation of the NLRP3 inflammasome. ^14^ While these natural peptides offer promising biological profiles, IDR-1002 provides distinct advantages, including synthetic accessibility, the modularity of its amino acid sequence for optimization, and a well-characterized immunomodulatory profile across diverse models. ^12^ ^,^ ^26^ ^,^ ^28^ ^,^ ^42^ Recent evidence underscores the growing importance of bioactive peptides as strategic tools to target Nrf2 for alleviating inflammation. ^67^ Specifically, in endothelial cell physiology, these peptides are now recognized as pivotal regulators at the mechanistic and pharmacological crossroads of vascular health. ^68^

The broader immunomodulatory activities previously described for IDR peptides help contextualize the differential Nrf2 responses observed in this study. Evidence from other innate defense regulator peptides supports the dual antioxidant and anti-inflammatory profile observed for IDR-1002. Studies in human neutrophils have shown that IDR-1018 and HH2 reduce ROS production, suppress TNF-α release, and promote LL-37 secretion, demonstrating that IDRs can simultaneously modulate oxidative and inflammatory pathways. ^17^ Additionally, IDR-1 has been reported to interact with the ZZ domain of p62/SQSTM1, a key regulator of the Keap1–Nrf2 axis, providing a mechanistic explanation on how certain IDRs may facilitate Nrf2 stabilization through p62-dependent sequestration of Keap1. ^69^

Despite that IDR-1002 induces a more pronounced Nrf2 nuclear accumulation than IDR-1 in the HEK293 cell model, the specific contribution of p62-dependent pathways or other upstream regulatory kinases for this particular peptide remains to be fully elucidated. In our model, the calculated *EC*_50_ for Nrf2-driven transcriptional activity was 18.57 μM, reflecting a nuanced potency in the reporter assay; however, the pronounced nuclear presence suggests that IDR-1002 effectively facilitates the initial stages of the antioxidant response. This Nrf2 nuclear accumulation, coupled with its previously reported NF-κB inhibition, supports the idea that IDR-1002 integrates both antioxidant and anti-inflammatory activities more effectively than other related synthetic peptides. These results indicate that while the peptide’s potency for gene induction is moderate, its capacity to modulate cellular redox sensors is notable. Although the precise molecular target of IDR-1002 that bridges these two nodes is currently under investigation, our functional data clearly position this peptide as a versatile dual-function regulator within the innate defense regulator family.

Although these findings are promising, further experimental validation is necessary to confirm direct binding between IDR-1002 and its biological target in order to determine if this interaction modulates Keap1-mediated degradation. Based on our previous finding that lactoferricin B-derived peptide (FKC) targets TNFR1, ^70^ and our recent observations that FKC also activates Nrf2, we investigated if IDR-1002 shared this mechanism. However, FRET-based monitoring of TNFR1 conformational dynamics showed no inter-monomeric changes, indicating that, unlike FKC, IDR-1002 is not a direct receptor antagonist or allosteric modulator. Consequently, its dual activity must involve an alternative receptor or signaling node, narrowing the search for its primary cellular target. A leading candidate for such a role is the Keap1-Nrf2 axis, which remains a premier target for therapy due to its central role in preventing chronic inflammatory and oxidative damage. ^71^ ^,^ ^72^ The recent development of high-affinity, selective inhibitors of the Keap1–Nrf2 protein-protein interaction (PPI) by Lin et al. (2025) provides a compelling structural precedent for such a possibility. ^73^

Given its specific size and cationic nature, it is plausible to suggest that IDR-1002 might act as a competitive or allosteric inhibitor of this PPI, potentially favored by the electrostatic affinity between the peptide’s positive residues and the anionic motifs of the Nrf2 Neh2 domain (specifically the DLG and ETGE). Under this hypothetical framework, IDR-1002 could potentially offer a more targeted and cytocompatible alternative for Nrf2 activation compared to classical electrophilic agents; by potentially avoiding the covalent modification of cellular thiols, the peptide is expected to minimize the risk of non-specific off-target effects. Nevertheless, further computational or biophysical validation remains essential to definitively characterize the binding kinetics of this interaction. Future studies utilizing Molecular Dynamics (MD), Surface Plasmon Resonance (SPR) or Isothermal Titration Calorimetry (ITC) will be required to confirm the physical binding constants and thermodynamic profile between the peptide and the specific Neh2 motifs, bridging the gap between our functional observations and the underlying molecular recognition. Although, the consistent correlation between Nrf2 nuclear translocation and the specific induction of downstream enzymes (HO-1, NQO1, GCLM) provides compelling evidence of the pathway’s activation by IDR-1002, we acknowledge that absolute Nrf2-dependence for the observed antioxidant protection remains to be definitively confirmed. Specifically, while IDR-1002 treatment resulted in a significant improvement in the intracellular redox state demonstrated by an enhanced capacity to neutralize H _2_O _2_ challenges, we recognize that proving this protection is exclusively Nrf2-dependent requires genetic silencing or chemical inhibition.

As an alternative we decided to determine a robust functional validation approach due to the significant biological confounding factors associated with Nrf2-deficient models. First, Nrf2-deficient cells exhibit profound mitochondrial instability and NADPH deficits, which in endothelial models like BECs, leads to hypersensitivity to routine handling and *‘handling-induced death*’. ^74^ Second, current pharmacological tools present significant specificity risks; ML385 has been recently highlighted for its cross-reactivity with structurally homologous factors like Nrf1 and CREB, ^75^ ^,^ ^76^ while Brusatol acts as a global translation inhibitor affecting multiple essential regulators like c-Myc, ^77^ making it difficult to discriminate Nrf2-specific effects. Moreover, stable knockdown via CRISPR or shRNA often triggers deep redox reprogramming and compensatory mechanisms (e.g., Nrf1 stabilization), ^78^ ^,^ ^79^ potentially deviating from the original physiological state of BEC cells. Future studies using transient, high-specificity interference strategies will be essential to conclusively link the molecular activation of Nrf2 to the global redox fortification observed in our BEC model.

Finally, despite the encouraging evidence presented here, a deep insight on the pharmacokinetic properties, bioavailability, and potential off-target effects requires further investigation. Additionally, validation of these findings in animal models of oxidative and inflammatory diseases are mandatory. In spite of these limitations, IDR-1002 emerges as a non-cytotoxic, dual-function synthetic peptide that simultaneously modulates Nrf2 and NF-κB signaling. Its capacity to co-regulate these pathways provides a valuable model for studying the interplay between cellular redox homeostasis and inflammatory signaling, potentially informing the future development of more targeted immunomodulatory strategies.

## Data Availability

Figshare: Files that contain data on Nrf2 activation by the cationic peptide IDR-1002. https://doi.org/10.6084/m9.figshare.31093330. ^80^
○ WB peptides IDR, densitometry data.xlsx (Data used to generate Figures 1A-D). ○ WB Nrf2 data analysis HEK293 cells; peptides IDR.pzfx (Data used for statistical analysis of Figures 1A and D). ○ Densitometry data WB in BEC cells with (IDR-1002).xlsx (Data used to generate Figures 1E and F; Figures 2B and C). ○ WB Nrf2 data analysis BEC cells; IDR-1002.pzfx (Data used for statistical analysis of Figures 1E and F). ○ HepG2 EC50 assay IDR-1002; raw and normalized data.pzfx (Data used to generate Figure 2A). ○ HO-1_NQO1_GCLM ELISA normalized data 2h and 4h.pzfx (Data used to generate Figures 2E-F). ○ GST activity assay 6,12,18,24 h; raw and normalized data.pzfx (Data used to generate Figure 3). ○ General intracellular oxidative stress assay; raw data and normalized.pzfx (Data used to generate Figure 4). ○ TNF alpha assay; raw and normalized data.pzfx (Data used to generate Figure 5). ○ Quantitative Assessment of Subcellular Fractionation Purity and Signal Correction data.xlsx Data are available under the terms of the Creative Commons Attribution 4.0 International license (CC-BY 4.0). Figshare: Files that contain the supplementary Figure S1 and data on IDR-1002 cytotoxicity. https://doi.org/10.6084/m9.figshare.31116373.v1. ^81^
○ MTT assay in Hep G2 cells 8 h; raw and normalized data.pzfx (Data used to generate supplementary Figure 1A). ○ MTT assay in bovine endothelial cells (BEC) 8 h; raw and normalized data.pzfx (Data used to generate supplementary Figure 1B). ○ Supplementary FIGURE S1_Romero-Duran-2026.tif Data are available under the terms of the Creative Commons Attribution 4.0 International license (CC-BY 4.0).
