# Supplementary material for: PEPTIDE IDR-1002 REGULATES THE ANTIOXIDANT AND ANTI-INFLAMMATORY RESPONSES BY ACTIVATING THE KEAP1-NRF2 SIGNALING PATHWAY
Source: F1000Res. 2026 Aug 4;15:204. Originally published 2026 Feb 6. [Version 4] doi: 10.12688/f1000research.177148.4 (PMC13560003; doi:10.12688/f1000research.177148.4)
Supplement: Peer review history

# Reviewer report: Reviewer response for version 4

**DOI:** 10.5256/f1000research.205663.r507800

**Published:** 2026-08-06

**Authors:** Carlos Rosales

**Affiliations:** 1 Universidad Nacional Autónoma de México, Mexico City, Mexico

Authors have revised their manuscript addressing all my previous concerns. This report is much clearer now and should indexed.

Is the work clearly and accurately presented and does it cite the current literature?

Yes

If applicable, is the statistical analysis and its interpretation appropriate?

Yes

Are all the source data underlying the results available to ensure full reproducibility?

Yes

Is the study design appropriate and is the work technically sound?

Yes

Are the conclusions drawn adequately supported by the results?

Yes

Are sufficient details of methods and analysis provided to allow replication by others?

No

Reviewer Expertise:

Immunology. Signal transduction

I confirm that I have read this submission and believe that I have an appropriate level of expertise to confirm that it is of an acceptable scientific standard.

# Reviewer report: Reviewer response for version 3

**DOI:** 10.5256/f1000research.203889.r490994

**Published:** 2026-06-29

**Authors:** Carlos Rosales

**Affiliations:** 1 Universidad Nacional Autónoma de México, Mexico City, Mexico

The manuscript by Marco Antonio Romero-Durán, et al., describes the immunomodulatory effects of a 12-residue cationic peptide, named innate defense regulator (IDR)-1002, on endothelial cells. The peptide IDR-1002 was identified from a library of bactenecin, an antimicrobial peptide found in bovine neutrophils, and authors have previously shown that IDR-1002 modulates inflammation in macrophages (RAW 264.7 cells) stimulated with lipopolysaccharide, TNFα, or IL-1β. The reduction in inflammation was due to inhibition of IκBα phosphorylation and the consequent blockage of NF-κB translocation to the nucleus. In this report authors expand their study characterizing the effects of IDR-1002 on the antioxidant response of endothelial cells. Because, the transcription factor Nrf2 (Nuclear factor erythroid 2-related factor 2) acts as a master regulator of redox homeostasis and anti-inflammatory signaling in several cells, this report explored whether IDR-1002 could activate Nrf2 leading to an antioxidant response. It is shown that the peptide IDR-1002 indeed was able to activate the Nrf2-mediated antioxidant response in both human and bovine endothelial cells.

 Experiments are performed correctly and data show that the peptide IDR-1002 induced activation and translocation of Nrf2 to the nucleus. Also, IDR-1002 enhanced the transcriptional activity of ARE (Antioxidant Response Element)-driven genes, leading to enhanced production of several antioxidant enzymes. Data clearly support authors' conclusions that the peptide IDR-1002 activates an antioxidant response via Nrf2 signaling.

 Manuscript is well organized and easy to read. However, the materials and methods section is incomplete and therefore, the study may not be interpreted correctly, and also cannot be reproduced satisfactorily.

 Next, I point out several parts of the manuscript that need clarification to improve the quality of this report.

 The description of the antibodies used is inconsistent and cumbersome. Sometimes the antigen is mentioned other times the type if IgG involved is presented. Thus, it is not clear what type of antibody is used and what is the corresponding antigen the antibody recognizes. Full name for each antibody, in addition to the catalog number should be provided. For example, "mouse β-actin" should be "monoclonal IgG1 against β-Actin"; GAPDH-IgG" should be "mouse monoclonal IgG1 against GAPDH"; "Histone H3 Rabbit" should be      "Rabbit monoclonal antibody against histone H3"; "rabbit NQO1" should be "Rabbit monoclonal antibody against NQO1", and so on for all other antibodies. Similarly, "antimouse IgG-BP-HRP" is confusing. This reagent is a mouse IgG Fc binding protein (m-IgG Fc BP) conjugated to horseradish peroxidase (HRP). It is not itself an antibody and thus it should not be listed as such. Given the full name of each antibody will help the reader understand the experiments without having to look for the product information based on catalog numbers.

 What is 1K medium? This should be described in more detail. Many readers are not familiar with this type of medium

 Similarly, the term "ARE-Hep-G2 cells" is incomplete and not accurate. Cells should be described with their full name "ARE Luciferase Reporter HepG2 Cell Line" Also, the parent cell line is "HepG2" not "Hep-G2". A consistent nomenclature should be presented throughout the manuscript.

 In cell culture section, it reads "National Autonomous University of Mexico (UNAM)". The name of the university is a proper name and should not be translated. Same way "John Brown" is not written as "Juan Café", the name of the university should be always in Spanish as "Universidad Nacional Autónoma de México".

 Also, what is HPV16 E6E7 oncogenes? These should be spelled out and a reference provided

 In luciferase assay, it indicates "IDR-1002 (final concentration: 0.5–300 μM)" but in MTT cell viability assay it reads "IDR-1002 (1–100 μM)". Also, in intracellular oxidative stress assay, it says "DR-1002 (1–50 μM)". It is not clear why the different concentrations were used in the various assays. This needs to be clarified both in the methods section and in the results section.

 In MTT assay, it says "50 μL of MTT solution (5 mg/mL in PBS, for a final concentration of 10% v/v)" This is confusing. No volume of cells is defined. Thus, dilution of MTT is not clear. It is better to indicate the amount of reagents as molar concentrations.

 In Western blotting section, it says "BEC cells were grown in 6-well plates to ~90% confluence, serum-starved for ≥4 h, and then treated with 1, 10, 25, or 50 μM IDR-1002 for 1 h or 4 h before lysis". Although the sequence of manipulations is clear, the reason for them is not. Why do cells have to be serum-starved first? Please clarify the reason for each step.  

 In ELISA assays, it reads "Briefly, 100 μL of standards or cell lysates were added to pre-coated plates along with 10 μL of Balance Solution". Also, "Samples were then incubated with 50 μL of HRP-conjugate for 1 h at 37°C, where endogenous antigens competed for conjugate binding sites". In addition, " TMB substrate was added for color development" This description is not clear. Much detail is needed to interpret the results correctly. What is "standards"? Please explain. What are the plates pre-coated with? Please explain. What is HRP-conjugate? Is this a secondary antibody coupled to peroxidase? If so, please give the full name. What is "endogenous antigens"? and how do they compete for "conjugate binding sites"? Please define all these terms so that the ELISA assay can be understood.

 The phrase "Results were inversely proportional to the target protein concentration and were normalized to total protein content for each sample" is also very confusing. Not clear how data were analyzed and what they meant. Please explain.

 RESULTS

 In figure 1, Nrf2 is detected in whole cell lysates but not cytosolic fractions. Why did this protein disappear?  Nrf2 should be present in similar amounts in whole and cytosolic fractions since it is mainly found in cytosol. Authors need to discuss this discrepancy.

 Figures 1E and 1F should indicate that "Nuclear Nrf2" is the protein shown. Please correct the y axis label accordingly.

 In figure 1F, gray bars indicate that both TBHP and IDR-1002 were used together. I do not think this is the case. Please explain and/or correct the figure.

 In "IDR-1002 induces Nrf2 transcriptional activity" section it reads "we evaluated Nrf2 transcriptional activity in HepG2 liver cells stably expressing an ARE- luciferase reporter. This cell line is..." The name of the transfected cell line, which is no longer HepG2 should be clearly indicated here. For example: "we evaluated Nrf2 transcriptional activity in HepG2 liver cells stably expressing an ARE- luciferase reporter. This cell line, named ARE-HepG2 cell, is..."

 Similarly, "Following stimulation of HepG2 cells with..." should be changed to "Following stimulation of ARE-HepG2 cells with ..."  The text also reads "Importantly, cell viability assays confirmed that IDR-1002 did not induce cytotoxicity neither in Hep-G2 or BEC cells at 1, 10, 25, 50, or 100 μM (Extended data Figure E1)" However, the figure is labelled as "supplementary Figure S1". Please correct accordingly to avoid confusion. Also "Hep-G2" should be "HepG2".

 In Figure 4 the label "Relative Intracellular Redox State" is vague. No definition of this term is provided. The graph actually shows ROS production quantified by DCFH-DA fluorescence. This should be indicated in the y-axis of the graph.

Is the work clearly and accurately presented and does it cite the current literature?

Yes

If applicable, is the statistical analysis and its interpretation appropriate?

Yes

Are all the source data underlying the results available to ensure full reproducibility?

Yes

Is the study design appropriate and is the work technically sound?

Yes

Are the conclusions drawn adequately supported by the results?

Yes

Are sufficient details of methods and analysis provided to allow replication by others?

No

Reviewer Expertise:

Immunology. Signal transduction

I confirm that I have read this submission and believe that I have an appropriate level of expertise to confirm that it is of an acceptable scientific standard, however I have significant reservations, as outlined above.

# Response

**Published:** 2026-07-06

**Authors:** Victor Manuel Baizabal Aguirre

**Affiliations:** CMEB-FMVZ, Universidad Michoacana de San Nicolás de Hidalgo, Morelia, Michoacán, Mexico

**MATERIALS AND METHODS**

**1. The description of the antibodies used...**

      Modified in text.

**2.** **What is 1K medium?**

      Composition has been detailed in text.

**3.** **Similarly, the term "ARE-Hep-G2 cells" is incomplete...**

      Modified in text.

**4.** **In cell culture section, it reads...**

      Modified in text.

**5.** **Also, what is HPV16 E6E7 oncogenes?**

      Modified in text and reference cited (30).

**6.** **In luciferase assay, it indicates "IDR-1002...**

      We appreciate the reviewer's attention to detail regarding the concentration ranges. The specific                 concentrations of IDR-1002 used in each experiment were optimized through preliminary pilot studies         and guided by previously published literature for these specific assay types. The range in the oxidative       stress assay (see Figure S1 at https://doi.org/10.6084/m9.figshare.32903237) was chosen to test               cellular stress at a wide range of concentrations from 1 to 300 mM. The broader range in the luciferase       assay (0.5–300 μM) was necessary to determine the ED _50_ reported. This EC _50_ value (18.57 mM) was         then used as a reference to test the functional effects of IDR-1002 in a concentration range from 1 to           50 mM. We have updated the manuscript to explicitly state the justification for these experimental               designs in the Methods and Results sections.

**7.** **In MTT assay, it says "50 μL of MTT solution...**

      We thank the reviewer for pointing out the lack of clarity in the original text. We have thoroughly revised       this section to explicitly define the initial culture medium volume (180 µL) and the exact volume of MTT       solution added (20 µL), ensuring a well-defined final total volume of 200 µL per well to preserve the             10% v/v protocol standard. Furthermore, we have calculated and included the precise molar                         concentration of the reagent in the well. Adding 20 µL of a 5 mg/mL stock solution to a total volume of         200 µL results in a final mass concentration of 0.50 mg/mL, which corresponds to a final molar                     concentration of 1.21 mM of MTT (molecular mass 414.32 g/mol). These specifications have been               updated in the revised manuscript.

**8.** **In Western blotting section, it says...**

      In the context of the Keap1-Nrf2 signaling pathway, serum starvation is a critical methodological step           because failing to remove the serum would introduce confounding variables that interfere with the               accurate assessment of Nrf2 activation. Serum contains a complex mixture of hormones, growth                 factors, and cytokines that may cross-activate, phosphorylate, or stabilize Nrf2. Moreover, serum                 naturally contains exogenous antioxidants (i.e., albumin) and free radical scavengers that may                     neutralize oxidative stress or bind directly to IDR-1002. This means serum would create a high,                   uncontrolled level of baseline Nrf2 activation. Therefore, serum starvation is vital to minimize                       background noise and ensure that Nrf2 activation is solely driven by IDR-1002.

**9.** **In ELISA assays, it reads "Briefly, 100 μL...**

      We thank the reviewer for requesting a more comprehensive and clear description of the ELISA assay         principles and components. The assay was performed using a commercial Human NFE2R2 (Human           Nuclear Factor Erythroid 2 Related Factor 2) ELISA kit (Cat. No. MBS744368, MyBioSource), which           operates under a solid-phase competitive enzyme immunoassay format. To clarify the specific                     terminology queried, first the standards consist of a series of six ready-to-use vials containing highly           purified recombinant human NFE2R2 at fixed, sequential concentrations (0, 0.5, 1.0, 2.5, 5.0, and 10           ng/mL) used to construct a standard calibration curve for sample quantification. Second, the microtiter         stripwell plates are pre-coated by the manufacturer with a specific polyclonal anti-NFE2R2 antibody.           Third, the HRP-conjugate is an NFE2R2-HRP conjugate (NFE2R2 antigen chemically linked to                   Horseradish Peroxidase) and fourth " *Endogenous antigens*" refer to the native, unlabelled NFE2R2             protein naturally present within cell lysates. Given that our study employs two distinct ELISA modalities       with opposing signal behaviors, we have expanded the Materials and Methods section to clearly                 delineate the data analysis process for both assay formats and to explicitly include these biochemical         definitions and mechanistic details.

**10.** **The phrase "Results were inversely proportional...**

      We acknowledge the reviewer’s point regarding the confusion caused by a single generalized                     statement. Given that our study employs two distinct ELISA modalities with opposing signal behaviors         we have expanded this section to clearly delineate the data analysis process for both assay formats. 1)       For the competitive ELISA (Nrf2) as detailed in the revised text, the assay principle relies on steric               competition between the endogenous nuclear Nrf2 and the NFE2R2-HRP conjugate for a limited                 number of antibody binding sites and 2) For the Double Antibody Sandwich ELISAs (HO-1, NQO1, and       GCLM) format the quantity of the reporter enzyme complex is directly and positively correlated with the       amount of target analyte bound. We have fully rephrased this explanation in the Materials and Methods       section to prevent any ambiguity.

**RESULTS**

**1.** **In figure 1, Nrf2 is detected in whole...**

     We thank the reviewer for this comment. The detection of Nrf2 in whole cell lysates accounts for the            total protein pool present in both the cytosolic and nuclear compartments. Under baseline conditions,          Nrf2 is predominantly localized in the cytosol, where it is continuously degraded. However, upon                  activation or oxidative stress, cytosolic Nrf2 escapes degradation and rapidly translocates into the                nucleus to induce target gene transcription. Therefore, the absence or reduction of Nrf2 in the cytosolic      fraction, paired with its robust detection in whole cell lysates, directly reflecting this stimulus-induced            nuclear translocation and a shift in the intracellular protein distribution.

**2.** **Figures 1E and 1F should indicate...**

     We agree with the reviewer’s observation and in accordance with your suggestion, we have corrected        the Y-axis labels for both Figure 1E and Figure 1F to explicitly read: "Nuclear Nrf2 (fold change, WB or        ELISA)". The corresponding figure legends and text in the Results section have also been updated to          ensure consistency.

**3.** **In figure 1F, gray bars indicate...**

     We thank the reviewer for this observation. We apologize for the typographical error in the (+) and (-)          sign at the bottom of the graph. This Figure has been thoroughly corrected to reflect the true                        experimental setup by changing the signs under the gray bars to minus (-) for TBHP. The figure legend        has also been modified to avoid any further confusion.

**4.** **In "IDR-1002 induces Nrf2 transcriptional activity" section...**

     Modified in text.

**5.** **Similarly, "Following stimulation of HepG2 cells...**

     Modified in text.

**6.** **In Figure 4 the label "Relative Intracellular Redox State" is vague...**

    We agree with the reviewer. As correctly noted, the assay specifically quantifies ROS production using       the DCFH-DA fluorescent probe. We have modified the Y-axis label of Figure 4 and now reads "ROS           production quantified by DCFH-DA fluorescence". Additionally, we have fully updated and expanded the     Materials and Methods section, as well as the Figure 4 caption, to explicitly clarify how the DCF                   fluorescence intensity directly reflects intracellular ROS levels.

# Reviewer report: Reviewer response for version 2

**DOI:** 10.5256/f1000research.198845.r479955

**Published:** 2026-06-12

**Authors:** Sinead O'Rourke

**Affiliations:** 1 Trinity College Dublin, Dublin, Ireland

The authors have failed to provide the uncropped blots for the revised figure 1A. There are no such files included in the supplemental data. Furthermore, the authors fail to explain in their rebuttal or methods section how the representative blots are cropped from replicates. As it currently is presented, the lamin control blot still appears to contain cropped sections. 

 This data is unsatisfactory for indexing and cannot be endorsed without the authors providing all uncropped blots for any and all blots included in the manuscript in the supplemental data. Moreover, this supplemental data should be presented as a supplemental figure that is clearly referenced in the text.

Is the work clearly and accurately presented and does it cite the current literature?

Yes

If applicable, is the statistical analysis and its interpretation appropriate?

Yes

Are all the source data underlying the results available to ensure full reproducibility?

Partly

Is the study design appropriate and is the work technically sound?

Yes

Are the conclusions drawn adequately supported by the results?

Partly

Are sufficient details of methods and analysis provided to allow replication by others?

Yes

Reviewer Expertise:

Immunology, Inflammation, Oxidative Stress, Regenerative Medicine, Innate Immunity

I confirm that I have read this submission and believe that I have an appropriate level of expertise to state that I do not consider it to be of an acceptable scientific standard, for reasons outlined above.

# Response

**Published:** 2026-06-12

**Authors:** Victor Manuel Baizabal Aguirre

**Affiliations:** CMEB-FMVZ, Universidad Michoacana de San Nicolás de Hidalgo, Morelia, Michoacán, Mexico

Dear Dr. Synead O'Rourke,

 I understand perfectly your decision because in the version 2 of our article does not appear listed the new results on Lamin western blots, in which we demonstrate that Lamin signal is the only observable one. You can access this and other results at https://doi.org/10.6084/m9.figshare.32002761. As you can see, we performed several repetitions and all of them show discrete and well-defined bands corresponding to Lamin and no other signal. We chose to crop the Lamin signal from the same blots we obtained the Nrf2 signals. Please revise our Figshare uploaded data. We will be happy to answer any question you may have. In the next few days, we will be working on the manuscript to cite these blots properly as you sensibly suggest.

# Reviewer report: Reviewer response for version 2

**DOI:** 10.5256/f1000research.198845.r479956

**Published:** 2026-05-07

**Authors:** Qinjian Zhao

**Affiliations:** 1 Chongqing Medical University, Chongqing, China

Authors revised the submission according to my last comments as much as possible, its seem to accept or consider for indexing in this form.

 Thanks

Is the work clearly and accurately presented and does it cite the current literature?

Partly

If applicable, is the statistical analysis and its interpretation appropriate?

Yes

Are all the source data underlying the results available to ensure full reproducibility?

Partly

Is the study design appropriate and is the work technically sound?

Partly

Are the conclusions drawn adequately supported by the results?

Partly

Are sufficient details of methods and analysis provided to allow replication by others?

Partly

Reviewer Expertise:

Immunogenetics

I confirm that I have read this submission and believe that I have an appropriate level of expertise to confirm that it is of an acceptable scientific standard.

# Response

**Published:** 2026-05-07

**Authors:** Victor Manuel Baizabal Aguirre

**Affiliations:** CMEB-FMVZ, Universidad Michoacana de San Nicolás de Hidalgo, Morelia, Michoacán, Mexico

We truly appreciate your recommendation for its acceptance and indexing.

# Reviewer report: Reviewer response for version 1

**DOI:** 10.5256/f1000research.195321.r459182

**Published:** 2026-03-14

**Authors:** Sinead O'Rourke

**Affiliations:** 1 Trinity College Dublin, Dublin, Ireland

In this research article, the authors aimed to investigate the capacity of innate defence regulator peptides to activate transcription factor, Nrf2. Specifically, they provide compelling findings for peptide, IDR-1002, and its ability to activate Nrf2, which in turn promotes both antioxidant and anti-inflammatory effects in bovine endothelial cells. While the results of this article can be considered a significant contribution to investigations of novel Nrf2 inducers, it is recommended that the authors add further detail to both the introduction and discussion of the article to strengthen the rationale of their chosen experimental model (HEK293 and BEC cells) to further underscore the significance of these findings. There are also several comments below which the authors should address. This article can be recommended for indexing following these revisions.

  

- As mentioned above, the introduction and discussion section would benefit from discussing the role of endothelial cells in oxidative stress/damage, to strengthen rationale of BEC cells as an experimental model.
- Similarly, results section should explain rationale for HEK cells and Hep-G2 cells being selected as experimental model in addition to BEC cells.
- I commend the authors for transparently sharing their densitometry data, but there are concerns that the blot in Figure 1A has been photo edited at the control sites for laminin as the image appears distorted. Can the authors please provide all the uncropped blots for their western results and clarify this question.
- Furthermore, regarding the blot presented in Figure 2 B-C, GAPDH is an inappropriate housekeeping control, given that Nrf2 induction has been observed to affect cell metabolism. Can authors provide an alternative house-keeping control, for example b-actin as used in Figure 1?
- Based on graphs presented in Figure 2, the authors cannot state that increased expression of HO-1, NQO1, and GCLM is time-dependent, as it appears there is no statistical significance between IDR-1002 2 hr and 4 hr treatment. Only significance between Control and Treated cells. It is recommended that the authors rephrase this.
- Can the authors provide more detail/explanation of their chosen time point for GST assays when 2 and 4 hrs were chosen for western blot analysis.
- The authors state that “Altogether, these results indicate that IDR-1002 exerts its antioxidant effects through the activation of the Nrf2 pathway.” While it is very likely that IDR-1002 promotes antioxidant effects through Nrf2 induction, authors should acknowledge this cannot be confirmed without inhibitor experiments. This should be included in the discussion section.
- The authors mention that IDR-1002 may offer advantages in specificity compared to previously established inducers of Nrf2. How are the authors assessing specificity to Nrf2? The authors should clarify whether this was assessed in their experiments, or else in the discussion describe future experiments which can do so.
- It is unclear the rationale for assessing TNF expression specifically in endothelial cells, what role do they have in incidence of infection/inflammation? Again, this would be strengthened by further details in the introduction/discussion section regarding the role of endothelial cells in oxidative stress/inflammation, as well as the significance of showing reduced TNF expression in endothelial cells. For example, is there clinical significance to this effect? How does this help?
- Can the authors provide more detail/explanation of their chosen 1 hr pre-treatment for Figure 5, as opposed to 4 hrs previously used in other experiments?
- It is recommended that the authors rephrase Figure 5 caption, as expression can be misinterpreted as gene expression. The authors should change this to production for better clarity.
- The authors state in their discussion that GST “remains” elevated, however, there was no significant changes at earlier timepoints, therefore authors should rephrase to say GST activity was most significant at 24 hrs suggesting prolonged cytotoxic effect compared to observed earlier expression of HO-1 etc…
- The statement regarding “well-characterised immunomodulatory profile” in the discussion requires references.

Is the work clearly and accurately presented and does it cite the current literature?

Yes

If applicable, is the statistical analysis and its interpretation appropriate?

Yes

Are all the source data underlying the results available to ensure full reproducibility?

Partly

Is the study design appropriate and is the work technically sound?

Yes

Are the conclusions drawn adequately supported by the results?

Partly

Are sufficient details of methods and analysis provided to allow replication by others?

Yes

Reviewer Expertise:

Immunology, Inflammation, Oxidative Stress, Regenerative Medicine, Innate Immunity

I confirm that I have read this submission and believe that I have an appropriate level of expertise to confirm that it is of an acceptable scientific standard, however I have significant reservations, as outlined above.

# Response

**Published:** 2026-05-06

**Authors:** Victor Manuel Baizabal Aguirre

**Affiliations:** CMEB-FMVZ, Universidad Michoacana de San Nicolás de Hidalgo, Morelia, Michoacán, Mexico

**These are the responses to the Reviewer 2.**

**1.** We appreciate the reviewer’s suggestion to further elaborate on the role of endothelial cells in oxidative damage to strengthen the rationale for our experimental model. We have expanded the Introduction and Discussion to highlight that Bovine Endothelial Cells (BECs) are not merely a structural barrier, but a primary metabolic and immunological interface highly susceptible to oxidative stress.

 In the context of IDR-1002 and the Keap1-Nrf2 pathway, we present the following arguments to strengthen the rationale of our experimental model, maintaining a clear distinction between observed data and theoretical inferences:

- Endothelial cells (ECs) function as the primary sensors of hemodynamic and chemical stimuli. By utilizing BECs, we demonstrate how IDR-1002 interacts with the interface responsible for regulating systemic-to-local inflammatory flux. The high metabolic activity of ECs essential for active transport and maintaining vascular tone—makes them an ideal model for studying antioxidant interventions. Based on contemporary signaling models, the robust activation of Nrf2 in this lineage is expected to support cellular defense mechanisms against the oxidative load inherent to vascular functions (Sapeta-Nowińska et al., 2025).
- Unlike other cell types, oxidative stress in ECs has been linked to direct structural consequences, specifically the degradation of tight junction proteins. The antioxidant response triggered by IDR-1002 in our study suggests a potential mechanism to assist in preventing the vascular permeability associated with chronic inflammation. Existing literature indicates that Nrf2 activation in the endothelium plays a vital role in vascular protection, which could, theoretically, prevent the degradation of structural proteins under oxidative challenge (Citrin et al., 2025; He et al., 2011).
- Since our study involves bovine cells, it is pertinent to emphasize that BECs are a gold-standard model for studying bovine-specific inflammatory conditions (e.g., mastitis or respiratory disease). This model provides a high-fidelity representation of the bovine vascular response (Cajero-Juárez et al., 2002), ensuring that our observations regarding IDR-1002 are theoretically translatable to veterinary applications and large-mammal vascular biology.
- Evaluating Nrf2 activation in this specific cell type is justified as it represents a critical site where antioxidant regulation could contribute to avoiding vascular hyperpermeability. Furthermore, the functional activation of the ARE-luciferase reporter observed in this study aligns with established master regulatory patterns of redox homeostasis and systemic detoxification hubs described in the literature (Bogen, 2017).

**References:**

 Cajero-Juárez M, Avila B, Ochoa A, et al.: Immortalization of bovine umbilical vein endothelial cells: a model for the study of vascular endothelium. *Eur. J. Cell Biol.* 2002; 81: 1–8.

 Sapeta-Nowińska M, Sołtys K, Gębczak K, et al.: Resistance of HEK-293 and COS-7 cell lines to oxidative stress as a model of metabolic response. *Acta Biochim. Pol.* 2025; 72: 14164.

 Citrin KM, Chaube B, Fernández-Hernando C, et al.: Intracellular endothelial cell metabolism in vascular function and dysfunction. *Trends Endocrinol. Metab.* 2025; 36: 744–755.

 He M, Siow RC, Sugden D, et al.: Induction of HO-1 and redox signaling in endothelial cells by advanced glycation end products: A role for Nrf2 in vascular protection in diabetes. *Nutr. Metab. Cardiovasc. Dis.* 2011; 21: 277–285.

 Bogen KT: Low-dose dose–response for in vitro Nrf2-ARE activation in human HepG2 cells. *Dose-Response.* 2017; 15: 1559325817737687.

**2. **We appreciate the reviewer's comment regarding the rationale for using multiple cell lines. To address this, we have clarified our selection criteria in the Introduction, Results and Discussion sections.

- While Bovine Endothelial Cells (BECs) represent the primary vascular interface of this study, HEK-293 cells were chosen as a gold-standard model to rigorously validate the molecular signaling of the Keap1-Nrf2 axis. This lineage has been recently characterized by its robust metabolic machinery and its reliability as a model for studying adaptive metabolic responses to oxidative stress (Sapeta-Nowińska et al., 2025). Their consistent phenotypic stability allows for a clear characterization of IDR-1002’s ability to trigger Nrf2 nuclear translocation, minimizing potential interference from cell-specific metabolic complexities.
- Furthermore, HepG2 cells were included to evaluate the peptide’s efficacy in a high-metabolic-demand environment. As the liver acts as a master regulatory hub for systemic detoxification and coordinates redox homeostasis through the Nrf2-ARE signaling pathway (Bogen, 2017), evaluating Nrf2 activation in this model suggests a potential for the peptide to modulate systemic cytoprotection.
- Together, the inclusion of HEK-293, HepG2, and BECs provides a comprehensive assessment of IDR-1002’s versatility. By demonstrating consistent activation of the Keap1-Nrf2 pathway across renal, hepatic, and vascular lineages, we propose that the peptide’s antioxidant and anti-inflammatory regulatory effects are part of a conserved, robust pharmacological mechanism rather than a cell-specific artifact. This cross-tissue validation strengthens the theoretical potential of IDR-1002 as a candidate for modulating oxidative stress-related conditions that could extend beyond the vascular system.

**References:**

 Sapeta-Nowińska M, Sołtys K, Gębczak K, et al.: Resistance of HEK-293 and COS-7 cell lines to oxidative stress as a model of metabolic response. *Acta Biochim. Pol.* 2025; 72: 14164.

 Bogen KT: Low-dose dose–response for in vitro Nrf2-ARE activation in human HepG2 cells. *Dose-Response.* 2017; 15: 1559325817737687.

**3. **We sincerely thank the reviewer for their careful inspection of Figure 1A and for the opportunity to address these concerns. Data integrity is a cornerstone of our research, and we take this observation very seriously.

 Regarding the original uncropped blots, we offer our most sincere apologies. Due to a hardware failure on the primary workstation where the high-resolution raw files were stored, we were unable to retrieve the original uncropped source images for the specific experiment shown in Figure 1A. To address your concern regarding the perceived distortion and to validate our findings, we performed new independent experiments using the same protein extracts from the original study. The following points clarify the situation:

- New Western blots for Lamin A/C (Laminin) were conducted in triplicate. Each replicate was processed on separate membranes to ensure consistency. The new densitometric analyses strictly confirm the original data, with no significant deviations in the observed trends.
- We have updated Figure 1A with a new, high-quality representative blot from these recent validation assays. We are now providing the uncropped, full-length blots for these new experiments as supplementary material to ensure total transparency.
- Regarding the original image, we categorically state that no intentional manipulation occurred. Lamin A/C is a high-molecular-weight protein (~70 kDa for Lamin A/C), and artifacts such as uneven transfer or background noise are common. The perceived " *distortion*" was likely a combination of mechanical membrane noise and global brightness/contrast adjustments applied to the entire blot to improve visibility.

We believe that providing these new, validated, and uncropped blots demonstrates our commitment to transparency and confirms that the numerical data shared is a faithful reflection of the biological phenomenon.

**4. **We completely agree with the reviewer’s concern regarding the potential metabolic interference of Nrf2 induction on GAPDH levels. To ensure the highest standards of normalization, we have replaced GAPDH with β-Actin as the loading control for Figure 2B–C. Detection of β-Actin was performed using the same protein extracts and, where possible, on the same membranes used for the target enzymes, by stripping or parallel blotting, to ensure a direct and accurate normalization. New densitometric analyses were performed for each enzyme, and the updated figures now reflect these more robust data, which fully confirm our initial findings with only minor variations.

**5. **We agree with the Reviewer’s observation that the absence of statistical significance between the 2-hour and 4-hour treatments precludes a definitive claim of a "time-dependent" progression. Accordingly, we have revised the manuscript to describe the effect of IDR-1002 as a "significant and sustained increase" in antioxidant enzyme production. While minor increments may be observed between 2 and 4 hours, the activation is consistently maintained at a stable plateau relative to the control across the evaluated timeframe. All references to time-dependence in this context have been removed for better clarity.

**Results section:**

 Treatment with IDR-1002 triggered a significant and sustained upregulation of the antioxidant genes HO-1, NQO1, and GCLM. Although the expression levels remained elevated from 2 to 4 hours post-treatment, no statistically significant difference was observed between these two time points, indicating that the induction reaches a plateau or remains stable following the initial response.

**Discussion section:**

 Our data demonstrates that IDR-1002 promotes the induction of Nrf2-dependent proteins. The increased production of HO-1, NQO1, and GCLM at both 2 and 4 hours suggests that, rather than eliciting a transient response, the peptide establishes a relatively stable antioxidant program within this time frame. This profile is consistent with a rapid activation of Nrf2 signaling followed by maintenance of downstream gene expression, indicating that IDR-1002 may effectively prime cellular defense mechanisms against oxidative stress. This pattern is particularly relevant in inflammatory contexts, where continuous antioxidant support is required to counterbalance persistent ROS production.

**6. **We thank the Reviewer for the opportunity to clarify the temporal design of our experiments. The temporal discrepancy between Western blot (2–4 h) and GST assays (18–24 h) reflects the biological transition from signaling initiation to functional metabolic reinforcement.

**1. Early Phase (2–4 h): Protein Expression of HO-1 and NQO1**

 Our objective at these time points was to capture the first wave of protein translation following Nrf2 nuclear translocation. As established by Kobayashi et al. (2006) and further supported by Senger et al. (2016), Nrf2 stabilizes rapidly after stimulation (such as with IDR-1002) due to the inhibition of its ubiquitination. Heme Oxygenase-1 (HO-1) and NAD(P)H:quinone oxidoreductase 1 (NQO1) are characterized as primary, rapid-response targets of Nrf2. Gu et al. (2011) demonstrated that while Nrf2 nuclear translocation is an immediate event, the subsequent protein upregulation of these early effectors is robustly detectable by Western blot within 2 to 4 hours. This early window allowed us to confirm that IDR-1002 successfully "switches on" the protective signaling machinery to mitigate immediate oxidative or inflammatory bursts (Huante-Mendoza et al., 2016; Madera & Hancock, 2012; Tonelli et al., 2018).

**2. Late Phase (18–24 h): Functional GST Enzymatic Activity**

 The rationale for extending the evaluation of Glutathione S-transferase (GST) (Hayes et al., 2005) activity to 18–24 h is based on the requirement for a significant expansion of the total cellular enzymatic pool. Unlike Western blot, which can detect the presence of a few protein molecules, functional assays measure the cumulative catalytic capacity of the cell (e.g., the conjugation of CDNB with glutathione). As demonstrated by (Leoncini et al., 2007; Angeloni et al., 2009) there is an intrinsic temporal " *delay*" in this process:

- The *de novo* synthesis of Phase II enzymes requires adequate time for ribosomes to process newly transcribed mRNA.
- GST proteins often accumulate more slowly due to their specific stability and metabolic turnover rates compared to HO-1.
- A higher threshold of protein accumulation is necessary to achieve a statistically significant increase in enzymatic activity.

While HO-1 and NQO1 act as the "first line of defense," the full expansion of the GST-mediated detoxification pool is a slower, cumulative process that typically requires 18–24 h to reach its maximal functional capacity.

**References:**

 Kobayashi M, Yamamoto M: Nrf2–Keap1 regulation of cellular defense mechanisms against electrophiles and reactive oxygen species. *Adv. Enzyme Regul.* 2006; 46: 113–140. doi:10.1016/j.advenzreg.2006.01.007.

 Gu J, Cheng Y, Wu H, et al.: Nrf2 nuclear translocation to upregulate phase II detoxifying enzyme expression coupled with the ERK, Akt and JNK signaling pathways. *Mol. Cell. Biochem.* 2011; 353: 101–109. doi:10.1007/s11010-011-0778-0.

 Tonelli C, Chio IIC, Tuveson DA: Transcriptional regulation by Nrf2. *Antioxid. Redox Signal.* 2018; 29: 1727–1745. doi:10.1089/ars.2017.7342.

 Senger DR, Li D, Jaminet SC, et al.: Activation of the Nrf2 cell defense pathway by ancient foods: disease prevention by important molecules and microbes lost from the modern Western diet. *PLoS ONE.* 2016; 11: e0148042. doi:10.1371/journal.pone.0148042.

 Madera L, Hancock REW: Synthetic immunomodulatory peptide IDR-1002 enhances monocyte migration and adhesion on fibronectin. *J. Innate Immun.* 2012; 4: 553–568. doi:10.1159/000336619.

 Hayes JD, Flanagan JU, Jowsey IR: Glutathione transferases. *Annu. Rev. Pharmacol. Toxicol.* 2005; 45: 51–88. doi:10.1146/annurev.pharmtox.45.120403.095857.

 Huante-Mendoza A, Silva-García O, Oviedo-Boyso J, et al.: Peptide IDR-1002 inhibits NF-κB nuclear translocation by inhibition of IκBα degradation and activates p38/ERK1/2–MSK1-dependent CREB phosphorylation in macrophages. *Front. Immunol.* 2016; 7: 533. doi:10.3389/fimmu.2016.00533.

 Angeloni C, Leoncini E, Malaguti M, et al.: Modulation of phase II enzymes by sulforaphane: implications for its cardioprotective potential. *J. Agric. Food Chem.* 2009; 57: 5615–5622. doi:10.1021/jf900549c.

 Leoncini E, Angeloni C, Malaguti M, et al.: Sulforaphane modulates phase 2 enzyme and Nrf2 expression in cultured rat cardiomyocytes. *Ital. J. Biochem.* 2007; 56: 101.

**7. **We thank the Reviewer for this critical observation. We agree that genetic silencing or chemical inhibition is a common approach to demonstrate pathway dependence. However, we have revised our manuscript to acknowledge this limitation and to clarify the rationale behind our functional validation strategy.

 While the consistent correlation between Nrf2 nuclear translocation and the specific induction of downstream enzymes (HO-1, NQO1, GCLM) provides marked evidence of the pathway's activation by IDR-1002, we recognize that absolute Nrf2-dependence for the observed antioxidant protection specifically the enhanced capacity to neutralize H _2_O _2_ challenges remains to be definitively confirmed.

 We emphasize that such experiments are necessary to confirm this molecular requirement; however, we opted for a robust functional approach due to significant biological confounding factors associated with current Nrf2-deficient models:

- In endothelial models like BECs, Nrf2-deficiency leads to profound mitochondrial instability and NADPH deficits, resulting in "handling-induced death" that can obscure specific peptide-induced effects (Holmström et al., 2013).
- Recent evidence highlights that ML385 carries risks of cross-reactivity with structurally homologous factors like Nrf1/CREB (Yan et al., 2024; Juszczak et al., 2024), while Brusatol acts as a global translation inhibitor (Harder et al., 2017).
- Stable knockdown (CRISPR/shRNA) often triggers deep redox reprogramming and compensatory mechanisms (e.g., Nrf1 stabilization), potentially deviating from the original physiological state of the BECs (Peretz et al., 2018; Deng et al., 2024).

In light of these challenges, we have updated the Discussion (Lines 655-664) to explicitly acknowledge that, while our data strongly support the involvement of Nrf2, additional studies employing transient, high-specificity approaches (such as inducible degron systems or biophysical binding assays including SPR and ITC) will be valuable to more directly link molecular Nrf2 activation with the broader redox fortification observed in our model.

**References:**

 Holmström KM, Baird L, Zhang Y, et al.: Nrf2 impacts cellular bioenergetics by controlling substrate availability for mitochondrial respiration. *Biol. Open*. 2013; 2(8): 761–770. DOI: 10.1242/bio.20134853.

 Harder B, Tian W, La Clair JJ, et al.: Brusatol overcomes chemoresistance through inhibition of protein translation. *Mol. Carcinog*. 2017; 56(5): 1493-1500. DOI: 10.1002/mc.22609.

 Yan L, Hu H, Feng L, et al.: ML385 promotes ferroptosis and radiotherapy sensitivity by inhibiting the NRF2-SLC7A11 pathway in esophageal squamous cell carcinoma. *Med. Oncol*. 2024; 41(12): 309. DOI: 10.1007/s12032-024-02483-6.

 Juszczak M, Tokarz P, Woźniak K: Potential of NRF2 Inhibitors-Retinoic Acid, K67, and ML-385-In Overcoming Doxorubicin Resistance in Promyelocytic Leukemia Cells. *Int. J. Mol. Sci*. 2024; 25(19): 10257. DOI: 10.3390/ijms251910257.

 Peretz L, Besser E, Hajbi R, et al.: Combined shRNA over CRISPR/cas9 as a methodology to detect off-target effects and a potential compensatory mechanism. *Sci. Rep*. 2018; 8(1): 93. DOI: 10.1038/s41598-017-18551-z.

 Deng R, Zheng Z, Hu S, et al.: Loss of Nrf1 rather than Nrf2 leads to inflammatory accumulation of lipids and reactive oxygen species in human hepatoma cells, which is alleviated by 2-bromopalmitate. Biochim. Biophys. *Acta Mol. Cell Res*. 2024; 1871(2): 119644. DOI: 10.1016/j.bbamcr.2023.119644.

**8. **We appreciate the Reviewer’s request for clarification on the term *'specificity*.' In our study, this refers to the hypothesis that IDR-1002 may act as a Protein-Protein Interaction (PPI) modulator of the Keap1-Nrf2 axis, distinguishing it from classical electrophilic inducers (e.g., sulforaphane) that rely on the non-specific covalent modification of Keap1 cysteine residues.

 Our suggestion of specificity is derived from the inherent physicochemical properties of IDR-1002. Given its cationic nature and specific 12-residue sequence, it is plausible that the peptide exhibits an electrostatic affinity for the anionic motifs within the Nrf2 Neh2 domain (such as the DLG and ETGE motifs). This potential interaction suggests a targeted displacement mechanism similar to recently developed non-electrophilic PPI inhibitors (Lin et al., 2025) which would avoid the systemic thiol-reactivity and collateral oxidative stress associated with traditional electrophilic agents.

 The following molecular coupling calculations may help to clarify our statement on specificity. These results, were not included in the manuscript, suggest IDR-1002 may interact with the Neh2 domain of Nrf2, blocking the natural interaction between the kelch domain of Keap1 and Nrf2.

 Peptide Docking calculations were carried out using the PIPER-FlexPepDock program, an ab-initio protocol designed to create high-resolution models of complexes between flexible peptides and globular proteins. PIPER-FlexPepDock is part of the well-known Rosetta Commons software. The results suggest a potential binding mode of the IDR-1002 at the low-affinity DLG motif within the Neh2 domain (Figure 1a). Additionally, we utilized AlphaFold-multimer to predict peptide-protein interactions at the Neh2 domain. In this case, the interaction also occurs at the low-affinity (DLG) Neh2 domain (Figure 1b).

 Note: Please find this Figure in the Figshare repository under Extended data.

 Taking together, both the PIPER-FlexPepDock protocol and AlphaFold-multimer predictions suggest a consistent peptide-protein interaction localized at the low-affinity DLG motif within the Nrf2 Neh2 domain. These findings provide structural support for the direct interaction between IDR-1002 and its target site.

 While our current experiments characterize the downstream activation of canonical Nrf2-target genes (HO-1, NQO1, GCLM), we acknowledge that absolute specificity against all other signaling pathways was not definitively quantified in this study. We have revised the Discussion to clarify that our focus on this pathway is based on these structural observations and the consistent activation of the Nrf2-ARE signaling (Lines 624-644).

**References:**

 Lin C, Narayanan D, Barreca M, et al.: Fragment-Based Drug Discovery of Novel High-affinity, Selective, and Anti-inflammatory Inhibitors of the Keap1-Nrf2 Protein-Protein Interaction. Angew. Chem. Int. Ed. Engl. 2025; 64: e202508121. doi:10.1002/anie.202508121.

**9. **We thank the Reviewer for this insightful comment regarding the rationale for assessing TNF-α expression in endothelial cells. In response, we have expanded the Introduction and Discussion sections (Lines 109-120 and 539-558) to better clarify the physiological and clinical relevance of this observation.

- Endothelial cells are now widely recognized as active participants in innate immunity, functioning as immunological sentinels at the interface between systemic circulation and tissues (Pober & Sessa, 2007). Upon stimulation, they produce pro-inflammatory cytokines such as TNF-α, which act in both autocrine and paracrine manners to induce the expression of adhesion molecules (e.g., ICAM-1 and VCAM-1) and chemokines that are essential for leukocyte recruitment and extravasation (Ley et al., 2007; Bradley, 2008). Thus, endothelial TNF-α represents a critical upstream regulator of inflammatory cell trafficking.
- Importantly, TNF-α also plays a central role in the regulation of endothelial barrier function. Elevated levels of this cytokine promote the disruption of intercellular junctions, leading to increased vascular permeability and facilitating the transition of inflammatory signals from the bloodstream into surrounding tissues (Mehta & Malik, 2006). This process is a hallmark of multiple inflammatory pathologies, including sepsis and chronic inflammatory diseases (Aird, 2007). Therefore, the observed reduction of TNF-α expression in endothelial cells following IDR-1002 treatment suggests a protective effect at the vascular level, limiting both leukocyte recruitment and vascular leakiness.
- Finally, the suppression of TNF-α provides functional support for the proposed crosstalk between the Nrf2 and NF-κB pathways. Activation of Nrf2 is known to negatively regulate NF-κB signaling, thereby reducing the transcription of pro-inflammatory mediators such as TNF-α (Wardyn et al., 2015; Ahmed et al., 2017). In this context, our findings indicate that IDR-1002-mediated activation of Nrf2 translates into a biologically relevant anti-inflammatory outcome in endothelial cells.

**References:**

 Pober JS, Sessa WC: Evolving functions of endothelial cells in inflammation. *Nat. Rev. Immunol.* 2007; 7: 803–815. doi:10.1038/nri2137.

 Ley K, Laudanna C, Cybulsky MI, et al.: Getting to the site of inflammation: the leukocyte adhesion cascade updated. *Nat. Rev. Immunol.* 2007; 7: 678–689. doi:10.1038/nri2156.

 Bradley JR: TNF-mediated inflammatory disease. *J. Pathol.* 2008; 214: 149–160. doi:10.1002/path.2287.

 Mehta D, Malik AB: Signaling mechanisms regulating endothelial permeability. *Physiol. Rev.* 2006; 86: 279–367. doi:10.1152/physrev.00012.2005.

 Aird WC: Phenotypic heterogeneity of the endothelium: II. Representative vascular beds. *Circ. Res.* 2007; 100: 174–190. doi:10.1161/01.RES.0000255690.03436.d3.

 Wardyn JD, Ponsford AH, Sanderson CM: Dissecting molecular cross-talk between Nrf2 and NF-κB response pathways. *Biochem. Soc. Trans.* 2015; 43: 621–626. doi:10.1042/BST20150014.

 Ahmed SM, Luo L, Namani A, et al.: Nrf2 signaling pathway: Pivotal roles in inflammation. *Biochim. Biophys. Acta Mol. Basis Dis.* 2017; 1863: 585–597. doi:10.1016/j.bbadis.2016.11.005.

**10. **We thank the Reviewer for the opportunity to clarify the experimental design. We would like to emphasize that the 1-h pre-treatment used in Figure 5 was strategically selected to evaluate the early priming and interference phase of the signaling response, whereas the 4-h periods in other experiments were intended to assess the late-stage accumulation of antioxidant enzymes.

 This 1-h incubation time is supported by three integrated molecular arguments:

- As established by Kobayashi et al. (2006), Nrf2 activation occurs via the rapid inhibition of its ubiquitination. This biochemical "switch" happens shortly after the initial peptide stimulation, allowing Nrf2 to accumulate in the nucleus within 60 minutes, placing the cell in a "pro-antioxidant state" prior to any further challenge.
- The work by Huante-Mendoza et al. (2016) demonstrates that IDR-1002 effectively inhibits NF-κB nuclear translocation by preventing IκBα degradation. Given the known crosstalk between NF-κB and Nrf2 where NF-κB-driven inflammation can mutually antagonize Nrf2 signaling a 1-h pre-treatment is critical. This ensures that the peptide blocks the pro-inflammatory machinery and promotes a cytoprotective environment before the secondary challenge (e.g., TNF-α) can trigger the NF-κB cascade.
- Consistent with Madera & Hancock (2012), IDR-1002 is a rapid immunomodulator that triggers signaling flux (such as PI3K-Akt and MAPK) within minutes. Choosing a 1-h window allows the peptide's signaling to reach its peak potency exactly when the secondary inflammatory challenge (10 ng/mL TNF-α) is introduced.

This is clearly evidenced in Figure 5, where the 1-h pre-treatment was sufficient to significantly decrease subsequent TNF-α production. This demonstrates that the peptide successfully "primed" the cells to interrupt the inflammatory feedback loop, a result that might be confounded by long-term metabolic adaptations if a 4-h window were used for this specific functional assay.

**References:**

 Kobayashi M, Yamamoto M: Nrf2–Keap1 regulation of cellular defense mechanisms against electrophiles and reactive oxygen species. *Adv. Enzyme Regul.* 2006; 46: 113–140. doi:10.1016/j.advenzreg.2006.01.007.

 Dinkova-Kostova AT, Kostov RV, Canning P: Keap1, the cysteine-based mammalian intracellular sensor for electrophiles and oxidants. *Arch. Biochem. Biophys.* 2017; 617: 84–93. doi:10.1016/j.abb.2016.08.005.

 Huante-Mendoza A, Silva-García O, Oviedo-Boyso J, et al.: Peptide IDR-1002 inhibits NF-κB nuclear translocation by inhibition of IκBα degradation and activates p38/ERK1/2–MSK1-dependent CREB phosphorylation in macrophages. *Front. Immunol.* 2016; 7: 533. doi:10.3389/fimmu.2016.00533.

 Wardyn JD, Ponsford AH, Sanderson CM: Dissecting molecular cross-talk between Nrf2 and NF-κB response pathways. *Biochem. Soc. Trans.* 2015; 43: 621–626. doi:10.1042/BST20150014.

 Ahmed SM, Luo L, Namani A, et al.: Nrf2 signaling pathway: pivotal roles in inflammation. *Biochim. Biophys. Acta Mol. Basis Dis.* 2017; 1863: 585–597. doi:10.1016/j.bbadis.2016.11.005.

 Madera L, Hancock REW: Synthetic immunomodulatory peptide IDR-1002 enhances monocyte migration and adhesion on fibronectin. *J. Innate Immun.* 2012; 4: 553–568. doi:10.1159/000336619.

 Niyonsaba F, Madera L, Afacan N, et al.: The innate defense regulator peptides IDR-HH2, IDR-1002, and IDR-1018 modulate human neutrophil functions. *J. Leukoc. Biol.* 2013; 94: 159–170. doi:10.1189/jlb.0213062.

**11. **We agree with the reviewer’s observation. To avoid any potential confusion with gene expression (mRNA levels), we have replaced the term "expression" with " **production**" throughout the Figure 5 caption and the corresponding text in the Results section. This term more accurately reflects the protein quantification performed by ELISA.

**12. **We appreciate the reviewer's precision regarding the enzymatic kinetics. We have rephrased the Discussion section to clarify that GST activity reached its peak significance at 24 h. This distinction highlights a sequential response, where the maximal induction of GST occurs later than the earlier production of HO-1 and NQO1, suggesting a prolonged protective or physiological effect.

**13. **We thank the Reviewer for pointing this out. We have updated the Discussion (Lines 583-584) to include the following key references that support the statement regarding the "well-characterised immunomodulatory profile" of IDR-1002 across different biological contexts:

 Nijnik A, Madera L, Ma S, et al.: Synthetic cationic peptide IDR-1002 provides protection against bacterial infections through chemokine induction and recruitment of neutrophils. *J. Immunol.* 2010; 184: 2539–2550. doi:10.4049/jimmunol.0901813. (Established the peptide’s role in providing protection against bacterial infections by modulating chemokine responses)

 Madera L, Hancock REW: Synthetic immunomodulatory peptide IDR-1002 enhances monocyte migration and adhesion on fibronectin. *J. Innate Immun.* 2012; 4: 553–568. doi:10.1159/000339324. (Characterized its effects on monocyte migration and adhesión)

 Huante-Mendoza A, Silva-García O, Oviedo-Boyso J, et al.: Peptide IDR-1002 inhibits NF-κB nuclear translocation by inhibition of IκBα degradation and activates p38/ERK1/2-MSK1-dependent CREB phosphorylation in macrophages. *Front. Immunol. *2016; 7: 533. (Demonstrated that IDR-1002 inhibits NF-κB nuclear translocation, a central mechanism in its anti-inflammatory profile)

 Sebők C, Pelyhe C, Giay L, et al.: Modulation of the immune response by the host defense peptide IDR-1002 in chicken hepatic cell culture. *Sci. Rep.* 2023; 13: 14530. doi:10.1038/s41598-023-41710-5. (Recently validated its immunomodulatory activity in hepatic cell models, supporting its relevance in metabolic tissues)

# Reviewer report: Reviewer response for version 1

**DOI:** 10.5256/f1000research.195321.r459174

**Published:** 2026-03-02

**Authors:** Qinjian Zhao

**Affiliations:** 1 Chongqing Medical University, Chongqing, China

1. The title overstates the mechanism by KEAP1–NRF2 pathway activation without direct evidence of Keap1 binding or disruption via molecular docking or another tools; the claim should be moderated.
2. Absent of Nrf2 knockdown or inhibition experiments were conducted, so Nrf2-dependence of the effects is not conclusively demonstrated.
3. NF-κB involvement is suggested, but no direct assays for activation of NF-κB (e.g., reporter assays or p65 translocation) are included.
4. Nuclear translocation data lack strong validation of fraction purity, weakening confidence in Nrf2 localization results.
5. ELISA measurement of Nrf2 does not validate the transcriptional activity or functional DNA binding.
6. The moderate potency was suggested according to reported EC50, yet the discussion presents IDR-1002 as a strong activator.
7. ROS assessment relies only on DCFDA, which is non-specific and prone to artifacts.
8. GST activity slightly declines at later time points, but the discussion frames it as sustained activation.
9. Mechanistic interpretations (e.g., p62 or upstream kinases) are speculative and not experimentally addressed.
10. Translational claims in conclusion are bit strong, but no in vivo experimental validation is provided.
11. The novelty is somewhat overstated given prior reports of IDR-1002’s anti-inflammatory effects.
12. Figures quality is not up to the mark.
13. References need to be updated.
14. Methodology needs to be cited where needed.
15. Overall current study offers compelling evidence that IDR-1002 activates Nrf2 signaling and reduces oxidative stress and inflammation in vitro. However, the manuscript lacks mechanistic insight into how IDR-1002 modulates the Keap1–Nrf2 axis, and causality has not been established through Nrf2 loss-of-function experiments. The claims regarding direct activation of the Keap1–Nrf2 pathway should therefore be moderated. Additional mechanistic studies would substantially strengthen the manuscript.

Is the work clearly and accurately presented and does it cite the current literature?

Partly

If applicable, is the statistical analysis and its interpretation appropriate?

Yes

Are all the source data underlying the results available to ensure full reproducibility?

Partly

Is the study design appropriate and is the work technically sound?

Partly

Are the conclusions drawn adequately supported by the results?

Partly

Are sufficient details of methods and analysis provided to allow replication by others?

Partly

Reviewer Expertise:

Immunogenetics

I confirm that I have read this submission and believe that I have an appropriate level of expertise to confirm that it is of an acceptable scientific standard, however I have significant reservations, as outlined above.

# Response

**Published:** 2026-05-06

**Authors:** Victor Manuel Baizabal Aguirre

**Affiliations:** CMEB-FMVZ, Universidad Michoacana de San Nicolás de Hidalgo, Morelia, Michoacán, Mexico

**These are the responses to the Reviewer 1.**

 1. We thank the reviewer for this comment and constructive suggestion and agree that that a more moderate title is appropriate for the current findings. Even though is it outside of the scope of this manuscript and prompted by your comment, we utilized computational techniques to assess the possibility of a direct interaction between the Neh2 domain of Nrf2 and the twelve-residue peptide IDR-1002.

 Peptide Docking calculations were carried out using the PIPER-FlexPepDock program, an ab-initio protocol designed to create high-resolution models of complexes between flexible peptides and globular proteins. PIPER-FlexPepDock is part of the well-known Rosetta Commons software. The results suggest a potential binding mode of the IDR-1002 at the low-affinity DLG motif within the Neh2 domain (Figure 1a). Additionally, we utilized AlphaFold-multimer to predict peptide-protein interactions at the Neh2 domain. In this case, the interaction also occurs at the low-affinity (DLG) Neh2 domain (Figure 1b).

 Note: Please find this Figure in the Figshare repository under Extended data.

 Taking together, both the PIPER-FlexPepDock protocol and AlphaFold-multimer predictions suggest a consistent peptide-protein interaction localized at the low-affinity DLG motif within the Nrf2 Neh2 domain. These findings provide structural support for the direct interaction between IDR-1002 and its target site.

 2. We thank you for this critical observation. We acknowledge that Nrf2 silencing is a standard approach; however, we deliberately prioritized robust functional validation over knockdown (KD) or chemical inhibition due to significant technical and biological confounding factors such as:

 Metabolic Artifacts (KD): Nrf2-deficient cells exhibit profound mitochondrial instability, NADPH deficits, and hypersensitivity to routine handling (trypsinization), which can lead to "handling-induced death" creating technical artifacts that obscure the specific effects of IDR-1002 (Holmström et al., 2013).

 Use of inhibitor ML385: Recent studies (Yan et al., 2024; Juszczak et al., 2024) highlight its risk of cross-reactivity with structurally homologous factors (Nrf1/CREB). Furthermore, its poor solubility requires high DMSO concentrations, which independently alter membrane fluidity and basal ROS.

 Use of inhibitor Brusatol: Acts as a global translation inhibitor, affecting essential regulators like c-Myc, making it impossible to attribute effects solely to Nrf2 (Harder et al., 2017).

 Stable knockdown with shRNA or CRISPR: These approaches often lead to the selection of "robust" clones that have activated compensatory mechanisms (e.g., Nrf1 stabilization or HSF1 activation) (Peretz et al., 2018; Rongzhen et al., 2024). The problem is that such genetically modified cells no longer accurately represent the original model, as they have undergone deep redox reprogramming to survive the loss of Nrf2.

 Comprehensive functional validation: To ensure data accuracy without these artifacts, we confirmed pathway activation through the following a alternative approaches:

 ARE-Luciferase Reporter Assay: Real-time monitoring of transcriptional activity without compromising the basal metabolic viability of the cells.

 Downstream Effectors: Dose-dependent induction of high-fidelity targets (HO-1, NQO1, GCLM).

 Enzymatic Activity: Confirmed a significant correlation between Nrf2 activation and a measurable induction of GST activity, which aligns with a general improvement in the intracellular redox state by showing an approximately 5.4-fold enhancement in the cellular antioxidant capacity.

 The consistent correlation between Nrf2 nuclear translocation, specific enzyme induction, and the resulting antioxidant effect provides a reliable representation of the Keap1-Nrf2-ARE axis activation by IDR-1002, avoiding the compromised data of inhibited or knockdown models.

 References

 Holmström KM, Baird L, Zhang Y, et al.: Nrf2 impacts cellular bioenergetics by controlling substrate availability for mitochondrial respiration. Biol. Open. 2013; 2(8): 761–770. DOI: 10.1242/bio.20134853.

 Harder B, Tian W, La Clair JJ, et al.: Brusatol overcomes chemoresistance through inhibition of protein translation. Mol. Carcinog. 2017; 56(5): 1493-1500. DOI: 10.1002/mc.22609.

 Yan L, Hu H, Feng L, et al.: ML385 promotes ferroptosis and radiotherapy sensitivity by inhibiting the NRF2-SLC7A11 pathway in esophageal squamous cell carcinoma. Med. Oncol. 2024; 41(12): 309. DOI: 10.1007/s12032-024-02483-6.

 Juszczak M, Tokarz P, Woźniak K: Potential of NRF2 Inhibitors-Retinoic Acid, K67, and ML-385-In Overcoming Doxorubicin Resistance in Promyelocytic Leukemia Cells. Int. J. Mol. Sci. 2024; 25(19): 10257. DOI: 10.3390/ijms251910257.

 Peretz L, Besser E, Hajbi R, et al.: Combined shRNA over CRISPR/cas9 as a methodology to detect off-target effects and a potential compensatory mechanism. Sci. Rep. 2018; 8(1): 93. DOI: 10.1038/s41598-017-18551-z.

 Deng R, Zheng Z, Hu S, et al.: Loss of Nrf1 rather than Nrf2 leads to inflammatory accumulation of lipids and reactive oxygen species in human hepatoma cells, which is alleviated by 2-bromopalmitate. Biochim. Biophys. Acta Mol. Cell Res. 2024; 1871(2): 119644. DOI: 10.1016/j.bbamcr.2023.119644.

 3. We appreciate this good observation. While NF-kB is a central mediator of inflammation, we prioritized Nrf2 signaling in this study based on the following mechanistic considerations:

 Previous experimental evidence reported: Our previous research established that IDR-1002 exerts its anti-inflammatory effects by inhibiting the activity of the IKK complex, preventing the phosphorylation and subsequent degradation of IκBα. By stabilizing IκBα, the peptide effectively sequesters NF-κB in the cytoplasm, blocking its nuclear translocation and DNA binding (Huante-Mendoza et al., 2016). This report demonstrated that reduction of pro-inflammatory cytokines, such as TNF-α, is mediated by the inhibition of upstream activation signals rather than direct antagonism of p65. Furthermore, IDR-1002 modulated the inflammatory response in similar contexts, primarily through the p38/ERK1/2-MSK1 signaling pathway, which triggered the phosphorylation of CREB. This activation promoted an immunomodulatory profile that worked in conjunction with the cytoplasmic sequestration of NF-κB. These findings support a mechanism where the reduction of pro-inflammatory cytokines, such as TNF-α, is a result of upstream signal interference and alternative transcriptional regulation, bypassing the need for direct intervention at the level of p65 nuclear translocation.

 Nrf2-NF-κB indirect crosstalk: Nrf2 activation exerts a potent indirect inhibitory effect on inflammation. By inducing antioxidant enzymes like HO-1, Nrf2 prevents the degradation of IkBα, thereby sequestering NF-kB in the cytoplasm (Huante-Mendoza et al., 2016; Gao et al., 2022). In this model, the reduction of TNFα is a downstream consequence of Nrf2-mediated redox stabilization rather than a direct antagonism of the NF-kB p65 subunit.

 In endothelial cells, the TNF promoter is not exclusively dependent on NF-κB (Pober, 2002). The transcriptional activation of TNF involves a coordinated enhanceosome complex that includes:

 AP-1 (c-Jun/c-Fos): Highly sensitive to the MAPK signaling that we have previously linked to IDR-1002.

 Ets-1 and Elk-1: Crucial for vascular inflammation and TNF-α induction.

 Redox Interference: Our results show a robust correlation between Nrf2 activation, Phase II enzyme induction (HO-1, GCLM, NQO1), and TNF-α reduction. This suggests that IDR-1002 acts via "redox-interference" where Nrf2 activation antagonizes the pro-inflammatory milieu.

 We have revised the Discussion to clarify that the observed reduction in TNF-α is a consequence of Nrf2-mediated antioxidant induction (Lines 539-558 and 559-571). In light of the evidence from our previous work (Huante-Mendoza et al., 2016) and the robust correlation between Nrf2 activation and Phase II gene expression (HO-1, NQO1, GCLM) shown in this study, we have prioritized Nrf2 signaling as the primary driver of the observed immunomodulatory phenotype. This interpretation replaces the previously suggested direct p65-mediated pathway. We now propose that the peptide promotes a redox-stable environment that indirectly antagonizes pro-inflammatory signaling, a mechanistic link that is more consistent with our current experimental data.

 References

 Huante-Mendoza A, Silva-García O, Oviedo-Boyso J, et al.: Peptide IDR-1002 inhibits NF-κB nuclear translocation by inhibition of IκBα degradation and activates p38/ERK1/2-MSK1-dependent CREB phosphorylation in macrophages stimulated with lipopolysaccharide. Front. Immunol. 2016; 7: 533. DOI: 10.3389/fimmu.2016.00533.

 Gao W, Guo L, Yang Y, et al.: Dissecting the crosstalk between Nrf2 and NF-κB response pathways in drug-induced toxicity. Front. Cell Dev. Biol. 2022; 9: 809952. DOI: 10.3389/fcell.2021.809952.

 Pober, JS. Activación endotelial: vías de señalización intracelular. Arthritis Res Ther 4, 2002 S109. https://doi.org/10.1186/ar576.

 4. We appreciate this important comment. We agree that demonstrating fraction purity is essential for an accurate interpretation of Nrf2 translocation data. To ensure the integrity of our results, we followed a rigorous and optimized protocol:

 Fractionation protocol: BEC cells were grown to ~90% confluence and serum-starved (≥ 4 h) prior to IDR-1002 treatment. To separate the compartments, we used the NE-PER Nuclear and Cytoplasmic Extraction Kit (Thermo Scientific), strictly following the manufacturer’s instructions to obtain high-purity fractions. Nuclear pellets were then lysed with 80 μL of RIPA buffer and sonicated using a Qsonica Q125 sonicator at 25% amplitude (3 cycles of 5 s) to ensure complete extraction of chromatin-bound proteins. This fractionation methodology is consistent with our previous work (Huante et al., 2016), where we successfully isolated highly pure cellular compartments to study MAPK-mediated signaling. In those studies, the same validation criteria were applied to confirm that peptide-induced changes were strictly compartment-specific, further supporting the reliability of our current findings.

 Purity monitoring and normalization: Fraction purity was monitored using high-fidelity loading controls: β-Actin (cytoplasm) and Lamin (nucleus). The absence of significant cross-reactivity confirmed the efficacy of the NE-PER kit in our cellular model. To prevent artifacts, nuclear Nrf2 levels were specifically normalized against Lamin, while cytoplasmic Nrf2 was normalized against β-Actin.

 Densitometric analysis and correction in Figure 1A: To ensure the highest precision in our findings, we performed a cross-contamination analysis for all lanes. Specifically for cases like IDR-1 and HH2, where minor traces of markers were detected in the opposite fraction, the Nrf2 signal was corrected by subtracting the proportional intensity attributed to technical contamination (see quantitative assessment of subcellular fractionation purity and signal correction). This mathematical normalization procedure ensures that Nuclear/Cytoplasmic ratio reported is a faithful reflection of the net compartmental distribution.

 A correction for compartmental mass transfer was applied. For each sample, a contamination coefficient (Fc) was calculated based on the nuclear marker (Lamin) signal in the cytoplasmic fraction. The net cytoplasmic signal of Nrf2 was obtained by subtracting the technical noise proportional to the nuclear pool:

 (Nrf2C_corr = Nrf2C - [Nrf2N * Fc])

 This approach ensures that the reported levels are biologically accurate and strictly represent the protein's localization. Regardless of technical variations in fractionation (marker carryover), the corrected densitometric analysis demonstrates that the peptides IDR-1002, 1018, IDR-1, and HH2 induce translocation of Nrf2 to the nuclear compartment. Furthermore, the mathematical cancellation of the residual cytoplasmic signal confirms that the pool of stabilized Nrf2 is predominantly located in the nucleus, thereby validating the activation of the Keap1-Nrf2-ARE antioxidant response pathway. Finally, the reliability of our fractionation procedure is the direct correlation between nuclear Nrf2 accumulation and the expression of these enzymes, which confirms that the protein detected in the nuclear fraction is functionally active.

 Reference

 Huante-Mendoza A, Silva-García O, Oviedo-Boyso J, et al.: Peptide IDR-1002 inhibits NF-κB nuclear translocation by inhibition of IκBα degradation and activates p38/ERK1/2-MSK1-dependent CREB phosphorylation in macrophages stimulated with lipopolysaccharide. Front. Immunol. 2016; 7: 533. DOI: 10.3389/fimmu.2016.00533.

 5. We appreciate your critical assessment regarding the functional implications of Nrf2 nuclear levels. While we agree that ELISA primarily quantifies protein abundance, we maintain that in our experimental model, the increase in nuclear Nrf2 is a high-fidelity surrogate for its transcriptional activation, based on the following integrated evidence:

 The gold standard for Nrf2 activity is the induction of its downstream effectors. Our data show a dose-dependent upregulation of HO-1, NQO1, and GCLM. Since these genes are strictly regulated by ARE-promoters, their induction confirms that the translocated Nrf2 (measured by ELISA) is functionally binding to DNA (Zhang et al., 2017). Our results mirror the activation kinetics of ARE-luciferase systems, where nuclear Nrf2 enrichment correlates linearly with enhanced promoter activity.

 The Keap1-Nrf2 signaling axis is primarily regulated at the level of protein stability and subcellular localization. Under the stimulus of IDR-1002, the disruption of the Keap1-Nrf2 interaction leads to the saturation of the nuclear compartment. Extensive literature confirms that once Nrf2 escapes cytoplasmic degradation and translocates to the nucleus, its heterodimerization with small Maf proteins and subsequent binding to the ARE sequence is the inevitable functional outcome.

 In summary, the ELISA-based detection of nuclear Nrf2 in our study does not stand in isolation; it is mechanistically linked to the robust induction of HO-1, GCLM, and NQO1. This 'triangulation' of data nuclear enrichment, canonical gene induction, and consistency with ARE-reporter models provides conclusive evidence of the transcriptional activity of Nrf2 induced by IDR-1002.

 Reference

 Zhang QY, Chu XY, Jiang LH, et al.: Identification of non-electrophilic Nrf2 activators from approved drugs. Molecules. 2017; 22(6): 883. DOI: 10.3390/molecules22060883.

 6. We thank the reviewer for this critical observation. While our results show that IDR-1002 induces a robust nuclear translocation of Nrf2, we agree that the term 'strong activator' should be adjusted to better align with the reported EC50 values, which suggest a more moderate potency. Consequently, we have revised the terminology to describe IDR-1002 simply as an activator, ensuring a more precise and objective representation of our findings while maintaining that the observed Nrf2 stabilization is a direct result of peptide-induced signaling.

 7. We thank you for this insightful comment regarding the inherent limitations of the DCFDA (2',7'-dichlorofluorescein diacetate) assay. We acknowledge that DCFDA serves as a general indicator of the cellular redox state rather than a specific sensor for individual oxygen radicals, such as hydrogen peroxide or superoxide (Deng et al., 2015).

 To address your concern and ensure the correct conclusion of our findings, we have refined our interpretation and methodology as follows:

 Terminological precision: We have revised the manuscript (see section: Assessment of General Intracellular Oxidative Stress in Materials and Methods) to refer to the observed changes as an increase in "general intracellular oxidative stress" or "overall redox disequilibrium" rather than attributing the signal to specific Reactive Oxygen Species (ROS).

 Control of artifacts: To minimize the risk of photo-oxidation and artificial redox cycling, all measurements were conducted under the following strictly controlled conditions.

 Background correction: As detailed in our methodology, fluorescence was corrected by subtracting signals from cell-free blank wells (to account for probe auto-oxidation) and untreated control cells (to account for cellular autofluorescence).

 Standardized protocol: Cells were incubated in the dark with 30 μM DCFH-DA for a consistent period (30 min) in phenol red-free conditions to reduce background interference.

 Data normalization: Results are now expressed as Relative Intracellular Redox State (Fold Change), ensuring that the signal reflects chemical oxidation rather than changes in probe concentration or cell density.

 Reference

 Deng R, Hua X, Li J, et al.: Oxidative stress markers induced by hyperosmolarity in primary human corneal epithelial cells. PLoS One. 2015; 10(5): e0126561. DOI: 10.1371/journal.pone.0126561.

 8. We thank the reviewer for this observation. We agree that the term 'sustained activation' should be refined to better reflect the experimental data. Regarding GST, while its activity remained elevated for much of the study, we observed a slight decrease in GST activity by the end of the 24-h incubation period. This suggests that while the peptide induces an initial antioxidant response, the enzymatic activation may undergo a gradual attenuation over prolonged periods. Since GSTs mediate the binding of glutathione to electrophilic compounds, promoting their clearance and reducing the cumulative damage caused by ROS and lipid peroxidation-derived products (Strazielle et al., 2025), their overall induction could provide a global defense against recurrent or prolonged oxidative stress episodes.

 Reference

 Strazielle N, Silva K, Rault E, et al.: The glutathione-dependent neuroprotective activity of the blood-CSF barrier is inducible through the Nrf2 signaling pathway during postnatal development. Fluids Barriers CNS. 2025; 22: 19.

 9. We sincerely thank the Reviewer for this insightful and critical observation. We fully agree that establishing the precise molecular interface between IDR-1002 and the Keap1–Nrf2 axis is a fundamental step for the complete characterization of this peptide. In accordance with your suggestion, we have moderated our mechanistic claims and expanded the Discussion to explicitly acknowledge these current limitations (Lines 597–609).

 Regarding the mechanistic interpretations, we have carefully rephrased our discussion of p62 and upstream regulatory kinases. Rather than presenting them as confirmed pathways for IDR-1002, we now describe them as potential regulatory nodes that warrant future experimental validation (Lines 597-599). This ensures a clear distinction between our solid functional observations and the theoretical signaling pathways that may be involved.

 10. We thank the Reviewer for this constructive critique regarding the translational language in our conclusions. We agree that the term "translational" should be used with caution, as our study is primarily focused on the molecular and cellular functional responses elicited by IDR-1002 in a bovine endothelial cell model.

 To ensure a balanced and scientifically rigorous interpretation of our findings, we have implemented the following changes:

 We have revised the concluding remarks (Lines 129-143, 559-571, and 662-667) to remove any definitive claims regarding clinical outcomes. Instead, we now emphasize that IDR-1002 acts as a "potential lead candidate" and that its ability to trigger the Keap1-Nrf2 axis provides a "functional rationale" for exploring its efficacy in more complex systems. While our current study is in vitro, the therapeutic interest in IDR-1002 is grounded in in vivo models of infection and inflammation (Turner-Brannen et al., 2011). Specifically, Nijnik et al. (2010) demonstrated that IDR-1002 significantly enhanced host protection in a mouse model of Staphylococcus aureus infection. In their study, the peptide reduced bacterial load and modulated the systemic inflammatory response without direct antimicrobial activity, highlighting its role as a potent Innate Defense Regulator (IDR). Our discovery that IDR-1002 promotes the induction of HO-1, GCLM, and NQO1 identifies Nrf2-mediated signaling as a candidate pathway that may contribute to the host protection observed in such studies, highlighting its role in neutralizing the pro-inflammatory milieu through redox stabilization. This evidence reinforces the potential of IDR-1002 as a promising translational candidate for the therapeutic management of complex inflammatory and oxidative disorders.

 References

 Nijnik A, Madera L, Ma S, et al.: Synthetic cationic peptide IDR-1002 provides protection against bacterial infections through chemokine induction and enhanced leukocyte recruitment. J. Immunol. 2010; 184(5): 2539–2550. DOI: 10.4049/jimmunol.0901813.

 Turner-Brannen E, Choi KY, Lippert DN, et al.: Modulation of interleukin-1β-induced inflammatory responses by a synthetic cationic innate defence regulator peptide, IDR-1002, in synovial fibroblasts. Arthritis Res. Ther. 2011; 13(4): R129. DOI: 10.1186/ar3440.

 11. We thank the Reviewer for this comment, which allows us to further clarify the unique contributions of our study. While it is true that the anti-inflammatory properties of IDR-1002 have been documented in previous literature, we have now demonstrated that this effect is driven by a dual molecular mechanism. IDR-1002 prevents the nuclear translocation of NF-κB by inhibiting IκBα phosphorylation and MAPK signaling (Huante-Mendoza et al, 2016), and simultaneously activates the Nrf2 signaling pathway.

 Of note, this study identifies Nrf2 as a critical regulatory node that complements the anti-inflammatory action of the peptide by reprogramming the cellular redox state. By demonstrating the subsequent induction of Phase II enzymes such as HO-1, NQO1, and GCLM, we define a critical molecular pathway through which this innate defense regulator (IDR) exerts its cytoprotective effects. Using a combination of nuclear translocation assays and functional assessments of the intracellular redox state (DCFH-DA), we prove that the anti-inflammatory and pro-antioxidant effects of IDR-1002 are intrinsically linked. This mechanistic detail provides a more holistic view of how the peptide manages cellular homeostasis under stress, effectively bridging the gap between active redox regulation and the modulation of the inflammatory response.

 In summary, while the observed outcome of reduced inflammation aligns with prior reports, the identification of the Keap1-Nrf2 signaling axis as a functional requirement is entirely novel. We have revised the Discussion sections (Lines 495-518) to more clearly articulate that this study identifies a new molecular 'node' in the peptide's signaling network. This finding effectively shifts the focus from simple cytokine inhibition to active redox regulation, providing a mechanistic link between the induction of antioxidant defenses and the modulation of inflammatory pathways.

 Reference

 Huante-Mendoza A, Silva-García O, Oviedo-Boyso J, et al.: Peptide IDR-1002 inhibits NF-κB nuclear translocation by inhibition of IκBα degradation and activates p38/ERK1/2-MSK1-dependent CREB phosphorylation in macrophages stimulated with lipopolysaccharide. Front. Immunol. 2016; 7: 533. DOI: 10.3389/fimmu.2016.00533.

 12. We appreciate your feedback regarding the visual presentation of our data. We take the clarity and quality of our scientific illustrations seriously to ensure the accurate communication of our findings.

 Importantly, all figures submitted with the manuscript were prepared according to the specific technical requirements of F1000Research, including resolution (minimum 600 DPI), and file format (TIFF/EPS). The Editorial Office conducted a preliminary technical screening and confirmed that the files met the journal's quality benchmarks for publication and peer review. Anyway, we decided to increase the resolution up to 1000 dpi for Figures 1 to 5 and 600 dpi for Figure S1.

 13. We appreciate the suggestion to update our bibliography. We agree that the field of IDR peptides and Nrf2-mediated redox signaling is rapidly evolving. Consequently, we have revised the manuscript to incorporate recent high-impact studies (2021–2025) that strengthen the conceptual framework of our study. In accordance with your suggestion, we have updated the references to include recent evidence that reinforces the mechanistic link between host defense peptides (HDPs) and Nrf2-mediated redox regulation:

 Notably, we included the study by Garg et al. (2024), which provides specific insights into how HDPs modulate endothelial cell physiology, and the review by Chu et al. (2025), which confirms the expanding role of bioactive peptides as potent Nrf2 activators for alleviating inflammation. To strengthen the mechanistic framework, we incorporated the reports by Garstkiewicz et al. (2017) and Mohan & Gupta (2018), which elucidate the non-transcriptional inhibition of the NLRP3 inflammasome and the essential crosstalk between TLR signaling and Nrf2, respectively.

 Furthermore, the clinical and pharmacological relevance of targeting the Keap1-Nrf2 axis is supported by the recent works of Adinolfi et al. (2023) and Chen et al. (2024). Finally, the inclusion of Lin et al. (2025) regarding high-affinity protein-protein interaction inhibitors provides a contemporary perspective on the potential of IDR-1002 as a non-electrophilic Nrf2 inducer. Collectively, these references position our findings within a modern pharmacological paradigm where Nrf2-mediated redox stabilization is recognized as a primary driver of immunomodulation.

 References

 Garstkiewicz M, Strittmatter GE, Grossi S, et al.: Opposing effects of Nrf2 and Nrf2-activating compounds on the NLRP3 inflammasome independent of Nrf2-mediated gene expression. Eur. J. Immunol. 2017; 47(5): 806–817. DOI: 10.1002/eji.201646665.

 Mohan S, Gupta D: Crosstalk of toll-like receptors signaling and Nrf2 pathway for regulation of inflammation. Biomed. Pharmacother. 2018; 108: 1866–1878. DOI: 10.1016/j.biopha.2018.10.019.

 Adinolfi S, Patinen T, Jawahar Deen A, et al.: The KEAP1-NRF2 pathway: Targets for therapy and role in cancer. Redox Biol. 2023; 63: 102726. DOI: 10.1016/j.redox.2023.102726.

 Chu Z, Zhu L, Zhou Y, et al.: Targeting Nrf2 by bioactive peptides alleviate inflammation: expanding the role of gut microbiota and metabolites. Crit. Rev. Food Sci. Nutr. 2025; 65(17): 3314–3333. DOI: 10.1080/10408398.2024.2367570.

 Garg VK, Joshi H, Sharma AK, et al.: Host defense peptides at the crossroad of endothelial cell physiology: Insight into mechanistic and pharmacological implications. Peptides. 2024; 182: 171320. DOI: 10.1016/j.peptides.2024.171320.

 Chen F, Xiao M, Hu S, et al.: Keap1-Nrf2 pathway: a key mechanism in the occurrence and development of cancer. Front. Oncol. 2024; 14: 1381467. DOI: 10.3389/fonc.2024.1381467.

 Lin C, Narayanan D, Barreca M, et al.: Fragment-based drug discovery of novel high-affinity, selective, and anti-inflammatory inhibitors of the Keap1-Nrf2 protein-protein interaction. Angew. Chem. Int. Ed. Engl. 2025; 64(39): e202508121. DOI: 10.1002/anie.202508121.

 14. We thank the Reviewer for the suggestion to enhance the technical transparency of our methodology. In the revised manuscript, we have integrated authoritative citations across all critical experimental stages to support the reproducibility of our findings. This includes the formal sourcing of cell lines and peptide synthesis standards, as well as the foundational protocols for subcellular fractionation and protein quantification. Furthermore, we have explicitly cited the mechanistic basis and known considerations of the DCFH-DA redox assay and the ARE-luciferase reporter validation, ensuring that our functional assays are anchored in established scientific literature. These references (detailed in the Materials and Methods section) provide the necessary detail to support the robust multi-tiered approach used to validate the Nrf2 signaling axis.
